# An analysis of oligomerization interfaces in transmembrane proteins

**DOI:** 10.1186/1472-6807-13-21

**Published:** 2013-10-17

**Authors:** Jose M Duarte, Nikhil Biyani, Kumaran Baskaran, Guido Capitani

**Affiliations:** 1 Laboratory of Biomolecular Research, Paul Scherrer Institut, Villigen, 5232, Switzerland

**Keywords:** Protein structure, Protein-protein interfaces, Membrane proteins, Eppic, Lipids, GPCR

## Abstract

**Background:**

The amount of transmembrane protein (TM) structures solved to date is now large enough to attempt large scale analyses. In particular, extensive studies of oligomeric interfaces in the transmembrane region are now possible.

**Results:**

We have compiled the first fully comprehensive set of validated transmembrane protein interfaces in order to study their features and assess what differentiates them from their soluble counterparts.

**Conclusions:**

The general features of TM interfaces do not differ much from those of soluble proteins: they are large, tightly packed and possess many interface core residues. In our set, membrane lipids were not found to significantly mediate protein-protein interfaces. Although no G protein-coupled receptor (GPCR) was included in the validated set, we analyzed the crystallographic dimerization interfaces proposed in the literature. We found that the putative dimer interfaces proposed for class A GPCRs do not show the usual patterns of stable biological interfaces, neither in terms of evolution nor of packing, thus they likely correspond to crystal interfaces. We cannot however rule out the possibility that they constitute transient or weak interfaces. In contrast we do observe a clear signature of biological interface for the proposed dimer of the class F human Smoothened receptor.

## Background

Transmembrane proteins (TMPs) play a central role in biology. They are responsible for some of the most important functions of cells like signalling, transport and catalysis of important reactions. As a consequence, large efforts have been directed at the structural and functional analysis of TMPs. This feat required a series of technical and conceptual advances ranging from a detailed understanding of TMP reconstitution, purification and crystallization in detergents to approaches for optimization of data collection and radiation damage mitigation at synchrotron light sources.

Those efforts were highly successful and the number of available TMP structures in the Protein Data Bank kept increasing exponentially since the first structure determination in 1985 [1]. The last 15 years witnessed structure determination breakthroughs in TMP families that had previously resisted all efforts, like G-protein coupled receptors and ABC-transporters. According to Stephen White’s MPSTRUC database of membrane proteins with known 3D structure (http://blanco.biomol.uci.edu/mpstruc), the number of unique membrane protein structures available as of 9 April 2013 is 393, a figure that includes not only TMPs but monotopic membrane proteins and some other membrane-associated proteins.

The abundance of high-quality structural data has made it possible to analyze membrane protein structures on a much larger scale and with a more solid foundation than only a few years ago. Studies have recently been performed on a variety of membrane protein-specific topics such as residue propensities at different membrane protein regions [2], lipid interactions [3], alpha-helical packing [4] or beta strand interactions [5].

This wealth of data makes it also possible to attempt a global analysis of protein-protein interactions and oligomerization in TMPs. To this end we compiled a manually curated dataset of membrane proteins for which the oligomeric state is well established from biophysical measurements and the structure has been determined at high resolution and quality. As analysis tool we used our Evolutionary Protein Protein Interface Classifier (EPPIC) [6], which we developed as a general approach to distinguish biological interfaces from lattice contacts in crystal structures. EPPIC depends on the availability of many homologues to the sequence of the protein being analyzed and its classification coverage and performance were retrospectively shown to improve, over a time span of 10 years, with the growth of the UniProt database. EPPIC reaches 90% accuracy on soluble proteins and we set out to assess its performance on our curated TMP dataset.

We also used our dataset to tackle an important issue in membrane protein structural biology: the presence and role of membrane lipids in TMP interfaces. The importance of lipids in membrane protein folding and oligomerization has been subjected to study in the last years [7-10]. We would like to ascertain whether structural evidence exists that provides any insights into the role of lipids in the oligomerization of TM proteins.

## Results and discussion

### The dataset

We compiled a dataset (TMPbio) of protein-protein interfaces that span the transmembrane region. In compiling such a dataset we adopted very strict selection criteria. First of all we restricted it to high resolution structures obtained from X-ray crystallography of 3-dimensional crystals in order to have a high quality and homogeneous dataset. The procedure required manual checking of the relevant literature to establish whether the oligomeric state of the TM proteins was known. Determining the oligomeric state of TM proteins experimentally is in itself a difficult task. Oligomerization can be measured in detergent via Size Exclusion Chromatography or Analytical Ultra Centrifugation as it would be the case for soluble proteins. However, the presence of detergent micelles and of the detergent belt around MPs complicates matters considerably. More sophisticated methods like FRET (Förster Resonance Electron Transfer) aim at determining the oligomerization state *in vivo* by using proteins tagged with chromophores and measuring the resonance energy transfer, very sensitive to distance [11,12]. Another *in vivo* approach exploits the dimerization-dependent transcriptional activation properties of *Vibrio cholerae* ToxR: chimeric constructs containing transmembrane segments of interest linked to ToxR can be quantitatively monitored for dimerization in an indicator strain [13-15].

Owing to the filtering criteria several important cases were excluded from this dataset:

•Bacteriorhodopsin: bacteriorhodopsin and archaeal rhodopsins form membranes in vivo (purple membrane) which can be considered as natural 2D crystals [16]. Crystallographic studies find them associated as trimers in the native environment [17,18]. However there is evidence of bacteriorhodopsin being a monomer in micelles [19] and even of it being functional in the monomeric state [20]. It was also solved via crystallization in bicelles [21] which resulted in a completely different crystal packing where no trimer association exists. Defining what constitutes an oligomer in the context of a 2D natural crystal thus becomes problematic. This precludes inclusion in the dataset since we need an independent non-crystallographic confirmation for the oligomerization state that it is not possible to provide for this case.

•GPCRs: there is a long-standing debate on class A GPCR oligomerization, see for instance [22-24]. Even though some experimental data are available and that some interfaces from crystal structures have been already proposed as possible dimerization interfaces [25-28] many questions remain open. Thus we decided not to include these interfaces in our dataset of *bona fide* biologically relevant TM interfaces. We did, however, study in detail the different proposed dimer interfaces, as described in the GPCR section below.

•Mitochondrial ADP/ATP carrier: despite it being initially characterized as dimer it was later proven to be a monomer [29,30] and thus the proposed lipid-mediated interface [31] was not included in this dataset. See also the Lipids and TM Interfaces section for further discussion.

The dataset comprises 62 oligomeric membrane protein structures with a total of 159 TM protein-protein interfaces, divided into the two subclasses: 46 from alpha class and 16 from beta class (see Additional files 1 and 2). This is, to our knowledge, the first fully comprehensive dataset of validated TM protein-protein interfaces from crystallography. All interfaces with their core residues can be easily visualized by inputting the corresponding PDB entry codes in our EPPIC web server (http://www.eppic-web.org) and looking at the output line corresponding to the interface Id. Additional file 1 provides direct links to the EPPIC results in the web server for each of the PDB entries.

We must note that the oligomerization state of the proteins in the dataset was most of the times assessed in a detergent-solubilized state. We cannot rule out the possibility that in some cases solubilization with detergents alters the protein association occurring in the cell. In any case it remains very difficult with current technologies to reliably assess membrane protein oligomerization *in vivo*. Hence, this analysis represents a best effort providing a snapshot of the current knowledge.

### Interface geometry and composition

**Figure 1:**
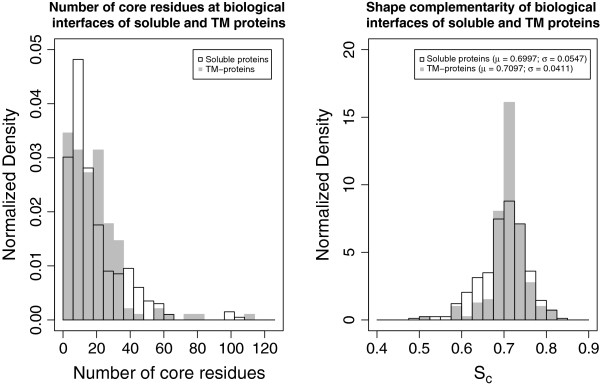
**Comparison of geometrical features in soluble and TM interfaces.** Left panel: distribution of interface core sizes (number of interface core residues) in both the soluble (black outline) and TM (gray) datasets. Right panel: distribution of shape complementarity indices (Sc) of TM interfaces (gray) and soluble protein interfaces (black outline).

The first analysis one can perform on the compiled dataset is in the geometry and composition of the interfaces. First of all we calculated the buried surfaces and number of interface core residues, which, as shown before for soluble proteins [6,32] are a strong indication of an interface to be biological. Additional file 1 presents the data for all interfaces. We compared the values for the TM interfaces with those of a composite dataset of soluble protein interfaces, obtained by merging the DCbio [6], PLP [32], Ponstingl dimer [33] and Bahadur dimer [34] sets. Overall the geometry is quite similar to that of soluble proteins with large interfaces (only 7 interfaces below 900 Å^2^) and many core residues (only 30 interfaces with 5 or fewer core residues, 15 with 4 or fewer). The left panel of Figure 1 presents the distribution of core sizes (number of core residues) for all interfaces in both soluble and TM interfaces, where it is apparent that in terms of number of core residues the TM interfaces do not differ much from their soluble counterparts. We then compared interface packing in TM and soluble interfaces, using their shape complementarity index (Sc) [35] as metrics. Again, the two groups of interfaces exhibited similar distributions for their Sc indices (right panel of Figure 1) indicating similarly tight packing. In summary, to form stable complexes, protomers need to come together forming tightly fitting surfaces with many buried “hot-spots” residues. It thus seems that the tight-packing requirement is not only a consequence of the water environment but that it is also necessary in the context of the lipid bilayer.

We found only a few exceptions to the above observation, almost exclusively limited to light harvesting and photosynthetic complexes. Those two protein complexes represent special cases since they contain a very large amount of chlorophylls and carotenoids. Their oligomerization interfaces are not strictly protein-protein but rather protein-cofactor-protein ones.

**Figure 2:**
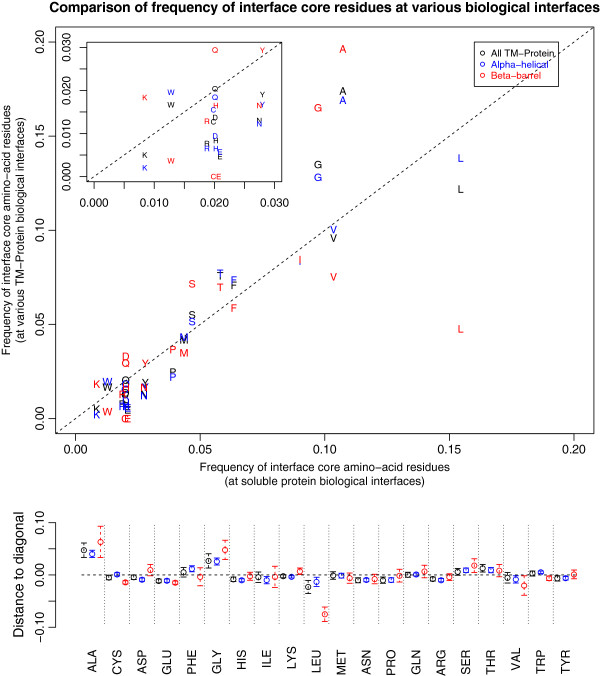
**Frequencies of the different amino acids in both TM protein interfaces and soluble ones.** The TM interfaces are further subdivided into alpha and beta classes. The inset in the top left is a magnification of the lower part of the plot. In the bottom panel we present the same data in terms of distance to the diagonal, including 95% confidence intervals estimated from bootstrapping. The only amino acids for which the confidence intervals are clearly out of the diagonal baseline (representing equal frequencies in soluble and TM) are alanine, glycine and leucine. Note that the scales of top and bottom plots are not the same.

Having confirmed that the packing of the TM interfaces is essentially like that of soluble ones, we studied whether any clear compositional differences in terms of the amino acid content can be observed. Figure 2 shows a comparison of amino acid frequencies at TM protein interfaces and at soluble protein interfaces. The membrane proteins are sorted into their two major structural classes: alpha and beta. It is apparent that in terms of amino acid composition membrane and soluble interfaces are also quite similar, with the exception of alanine and glycine for the alpha class and additionally leucine for the beta class. The first two residues are clearly overrepresented in TM interfaces compared to soluble ones, while leucine is underrepresented especially if one compares beta TM interfaces and soluble proteins. Constraints imposed by helical packing are a possible basis for this overrepresentation. It is known that in alpha helical TM domains small amino acids are important to enable helix packing [36,37]. Overrepresentation of Ala and Gly is less obviously connected to the subunit packing of beta TM proteins. We hypothesize that the flat interfaces formed by beta-to-beta packing also constrain the amino acids at the interface to be small as well as hydrophobic. A proposed reason for Gly overrepresentation in helix-helix packing is the favorable hydrogen bonding configuration of these residues in alpha-helices [38]. This could be indeed important for stability but might not be the main underlying cause, since Gly is also clearly over-represented in beta TM interfaces.

**Figure 3:**
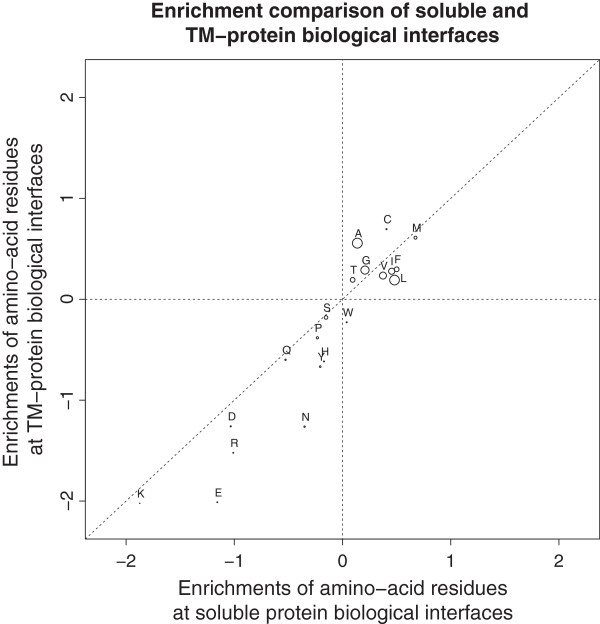
**Enrichments of amino acids in both TM protein interfaces and soluble protein interfaces.** Enrichments are defined as log-odd ratios of amino acid frequencies at interface core versus the rest of the protein. The size of the dots represents the averaged frequencies of amino acids at interface cores of both soluble and membrane protein sets, corresponding to the values in Figure 2.

**Figure 4:**
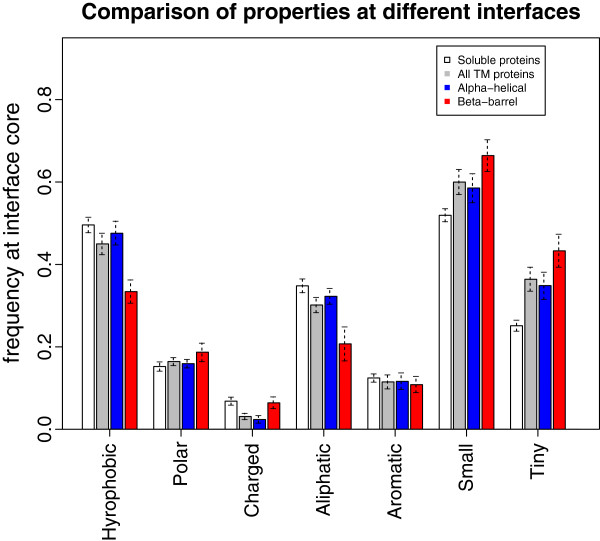
**Frequencies for the different groups of amino acids in interface core residues for either interface set: soluble proteins and TM proteins (further subdivided into alpha and beta classes).** See Methods for the amino acid properties grouping. 95% confidence intervals estimated through bootstrapping are given for each bar.

The data can also be presented in term of enrichments of the interface core residues versus the full protein (Figure 3) for both TM and soluble interfaces (see Methods for enrichment definition). The enrichments for most hydrophobic residues are clustered in the upper right quadrant while most charged or polar residues are clustered in the lower left quadrant. Thus for both soluble and TM interfaces the interface core residues are enriched in similar ways. Especially surprising is that no significant difference in enrichment can be seen for the hydrophobic residues in TM interfaces (alpha + beta) compared to soluble ones. This can be seen in a clearer way in Figure 4, where different properties of amino acids present at the interface cores are compared between the two groups of membrane and soluble proteins. Only if beta TM interfaces are considered alone the difference in hydrophobic amino acid frequencies appears to be clearly significant.

### Lipids and TM interfaces

We then set out to determine whether membrane lipids act as mediators in TM interfaces in our dataset. Lipid stoichiometry at the intramembranous surface of TM proteins is linked to the TM protein structure and degree of oligomerization [39]. The related concept that lipids can mediate certain TM protein interactions is also present in the literature [17,18,31] and is the subject of computational studies [10]. Hovewer, we were not able to find any significant membrane lipid-mediated TM interface in the entire validated dataset. This is in agreement with what was found above in the packing analysis. All interfaces present in the dataset are tightly packed, not leaving enough room for significant lipid interactions in the interfacial space. The case of the electron transport megacomplexes deserves to be discussed in some detail. The cytochrome bc1, cytochrome c oxidase and Photosystems I and II are possibly the most complicated of the known TM protein structures in terms of subunit content, size, topology and lack of symmetric features. The interfaces present in these structures are in many cases not purely TM but spanning both the soluble and TM regions. Additionally, as is the case with light harvesting complexes, the presence of many porphyrin-based cofactors adds to the complexity. Some lipids are seen in the interfacial spaces, for instance in the cytochrome bc1 complex [40] [PDB: 1ppj] a phosphatidylethanolamine molecule sits in a cavity where it interacts with chains C, D, E and J. However, the interaction of these chains occurs also through several extensive contacts on both intracellular and extracellular sides of the membrane.

Another interesting case is that of the bovine mitochondrial ADP/ATP carrier, where it was hypothesized that membrane lipids were essential for the interface formation. Initially it was characterized as a dimer [41]. Its first crystal structure [PDB: 1okc] [42] did not exhibit any plausible dimerization interfaces, since all of the crystal interfaces where either in an upside-down or head-to-tail orientation. Later on a new crystal structure was solved [PDB: 2c3e] where a very small interface (220 Å^2^) mediated by cardiolipins was proposed as the dimerization interface, though the authors recognized that further experimental support was required [31]. The case was finally settled by Bamber et al., who demonstrated in two separate papers that the carrier is actually a monomer in detergent [30] and that it also functions as a monomer *in vivo*[29].

The case of bacteriorhodopsin, which we did not include in the dataset as discussed above, also deserves mentioning. A belt of lipids is seen in the high resolution crystal structures of bacteriorhodopsin from Lipidic Cubic Phase (LCP) 3-dimensional crystals [PDB: 1m0k] [43], some of them located in the inter-trimer space. However the structure of a bacteriorhodopsin [PDB: 1kme] crystallized from bicelles [21] exhibits neither the trimeric arrangement nor the mediating lipids.

An important issue with membrane lipids is their high mobility and conformational flexibility, which makes it difficult to study them at atomic detail with crystallography. Indeed many of the crystallographic reported membrane lipids exhibit regions lacking electron density, which sometimes affects the interpretation and positioning of the entire ligand. In cases where chemically similar lipidic and detergent molecules are present in the crystal and ligand electron density is patchy it may even be challenging to distinguish a lipid from a detergent molecule. These issues belong to the broader problem of accurate electron density interpretation for non-protein ligands [44], which is often a challenge especially at the low resolution ranges typical of TM proteins. Independent validation for many ligands in the PDB has been performed and deposited in the Twilight server [44], where the ligand validity was objectively measured with a real space correlation coefficient (RSCC). Additional file 3 shows some prominent examples of Twilight RSCC values for lipids present in 11 representative alpha membrane proteins. Represented groups are bacteriorhodopsins, rhodopsins, potassium channel, ADP/ATP carrier, electron transport complexes, photosystems and light harvesting complexes. Out of 120 lipid molecules, 24 (20%) are below the Twilight threshold of RSCC 0.6, while 33% are below RSCC 0.7.

The above evidence speaks against a widespread role of lipids as mediators of biological protein-protein contacts, at least in the range of interface area covered by our TMPBio dataset. However, lipids can be essential crystallization agents. It has been shown that for a membrane protein to be able to crystallize in a LCP mesophase, the lipidic composition of the cubic phase is key to obtain crystals [45]. Not only the hosting lipids that form the bulk of the mesophase are important but in some cases also adding “doping” lipids like cholesterol is necessary for a successful crystallization [46].

### Classifying the interfaces with EPPIC

Once our dataset was compiled we used the method developed in our group [6] to attempt to computationally classify the TM interfaces as biologically relevant or not, as we previously did for soluble proteins. The EPPIC (Evolutionary Protein-Protein Interface Classifier) method relies on a combination of a simple geometrical indicator and of two evolutionary ones in order to classify an interface into biologically relevant or crystal lattice contact. It was demonstrated to work well on two validated sets of soluble proteins with an accuracy close to 90%.

Results for the TMPbio dataset are presented in Additional file 1, which also contains direct links to visualize results in full detail with the EPPIC web server. The overall classification accuracy for this ensemble of *bona fide* biological interfaces is 80% (127 out of 159 correctly classified), thus lower than that obtained earlier for soluble proteins [6]. It is worth mentioning that, in its current implementation, EPPIC analyzes interfaces in a pairwise manner only, without looking at the global assembly of interfaces present in the crystal and thus without taking the symmetry of the assembly into account. The symmetry of the assembly is indeed a very important factor, especially in membrane proteins where many of the known TM oligomers show highly symmetrical arrangements.

An example where the classification fails is in the structure of the rotor ring of Na-dependent F-ATP synthase [PDB: 2wgm]. The biological unit of this protein is a highly symmetric assembly with C11 point group symmetry, where chains consisting of a helical hairpin repeat 11 times around an axis. The core versus surface indicator cannot produce a prediction because of the few surface residues that are not interacting with other protomers. At the same time the rims of the interfaces happen to be very well conserved, possibly because some of the rim residues are involved in the sodium ion coordination. This results in high core versus rim values that fall out of the biological cut-off. The related structure of the rotor ring of a proton-dependent ATP synthase [PDB: 2wie] is misclassified by EPPIC in a very similar way, with analogous causes. The EPPIC method is known to have issues with small chains with little free surface like these cases. However the highly symmetric assembly of both cases would make a prediction based on symmetry considerations quite straightforward.

### GPCR oligomerization

Oligomerization of G protein-coupled receptors is one of the most heavily debated topics related to TM interfaces [22,47]. GPCRs constitute one of the largest protein families in animal genomes and are involved in receptor sensing and signal transduction processes, constituting one of the prime drug development targets with as much as 40% of drugs in the market targeting GPCRs. All members of the family share a very well conserved fold of 7 transmembrane helices and have evolved very fine selectivities in signal transduction. The family has been subdivided into 6 classes (class A to class F), being the class A of rhodopsin like receptors by far the most populated.

Most of the oligomerization debate has centered around the class A members where the evidence for oligomerization is least convincing [22]. In contrast it is quite well established that class C receptors exist as stable dimers [48]. Unfortunately no structure of the TM domain of a class C receptor is available to date. Experimentally, FRET techniques have repeatedly been used for establishing association of receptors in the membrane. For instance evidence from FRET exists for some class A receptors, like the CXCR4 receptor which was shown to homodimerize or heterodimerize with the CCR2 receptor [49,50].

Some dimer interfaces found by inspection of crystal structures have been proposed so far for several GPCRs [25-28]. Distinguishing relevant interfaces in crystal structures is indeed a non-trivial task, which has been subject to a large amount of investigation [6,51-54]. We decided to test the different proposed interfaces with the EPPIC method, which in principle is quite agnostic to crystallization artifacts, since it uses evolution to judge the biological relevance of an interface. The method is more powerful if abundant, relatively close sequence homologs are available for the alignments [6], especially if the distribution of identities in the homologs is uniform enough. Thus this makes the GPCR case a very suitable target for analysis with EPPIC, since sequence data are abundant for most family members. Predictions for this kind of case are *a priori* of a higher confidence.

We thus analyzed the different proposed interfaces (see Table 1):

•Bovine rhodopsin [PDB: 2i35, 2i36, 2i37] [26]: two crystal forms were solved in the study, both containing a similar dimer interface. The trigonal crystal form has 3 molecules in the asymmetric unit and the dimer interface appears twice in that form, once between monomers A + B and another time between 2 symmetry-related C monomers. The buried surface area of the different dimers ranges from ~300 Å^2^ to up to ~700 Å^2^, which is quite a significant variation, maybe attributable to the low resolution of the structures. In any case for all of them the packing in terms of number of core (fully buried) residues is typical for crystal contacts, ranging from 0 to 2 core residues counting both sides of the interface. The EPPIC evolutionary indicators, based on a large alignment of 105 homologs within 60% identity, also suggest a crystal contact in all cases, even though in some of them poor packing does not allow the program to make a decision, as EPPIC requires at least 8 residues buried to 70% in order to produce a prediction.

•It must be noted that the structures were determined at fairly low resolution: 3.7 Å, 4.1 Å and 4.2 Å, respectively. In that range of resolution it is quite difficult or impossible to properly model side chain rotamers, which may affect the packing quality of interfaces.

•Human CXCR4 chemokine receptor [PDB: 3odu, 3oe0, 3oe6, 3oe8, 3oe9] [27]: five receptor structures, bound to a small-molecule antagonist or to a cyclic peptide, were solved in several crystal forms. The crystallization constructs were engineered for stability by insertion of a T4 lysozyme between TM helices V and VI. This way the lysozyme molecule becomes a soluble “domain” of the receptor. A dimerization interface can be seen in all of them in a parallel arrangement with poor packing (no core residues at all). The artificially inserted lysozyme “domain” is involved in some of those interfaces, which accounts for their larger size. We analyzed the evolutionary signal of the interfaces by stripping off the lysozyme from the atomic model and found a consistent crystal contact signature for all of them.

•Human κ-opioid receptor [PDB: 4djh] [25]: the receptor was crystallized by engineering a T4 lysozyme fusion protein. An interface of 1000 Å^2^, in which the lysozyme is not involved, was proposed as dimerization interface. In terms of packing the interface features the typical signature of crystal contacts with few core residues (only 2). Evolutionary analysis by EPPIC again yields a very clear crystal contact signal, based on an alignment of 106 homolog sequences within 60% identity of the human κ-opioid receptor.

•Turkey β1 adrenergic receptor [PDB: 4gpo] [28]: in this case the crystallization strategy did not involve engineering of a fusion protein, but a set of stabilizing mutations plus removal of a loop. An interface of 800 Å^2^ between NCS-related chains A and B was proposed to mediate receptor dimerization. Evolutionary analysis again indicates a clear crystal contact, based on an alignment of 71 homologs. Again it must be noted that the structure was solved at fairly low resolution.

**Table 1:** The analyzed GPCR interfaces

**PDB**	**Chains**	**BSA**	**Cores 95%**	**Cores 70%**	**# seqs.**	**core-rim**	**core-surface**
2i35	A + A	316.6	0 + 0	2 + 2	105	0.39*	-0.20*
2i36	C + C	684.5	1 + 1	1 + 1	105	0.47 (b)	-0.35 (x)
	A + B	509.9	1 + 1	2 + 2	105	1.08*	0.50*
2i37	A + B	418.2	0 + 0	2 + 2	105	0.45*	-0.23*
	C + C	413.2	0 + 0	2 + 2	105	0.41*	-0.25*
3odu	A + B	1209.3(797.9)	0 + 0		52	1.28 (x)	1.55 (x)
3oe0	A + A	1089.4(1086.8)	0 + 0		83	1.55 (x)	1.71 (x)
3oe6	A + A	1037.6(764.8)	0 + 0		94	1.67 (x)	2.80 (x)
3oe8	B + C	665.2(591.9)	0 + 0	3 + 4	83	0.83*	0.32*
3oe9	A + B	959.4(877.4)	0 + 0		102	1.46 (x)	1.70 (x)
4djh	A + B	1024.0	1 + 1		106	1.42 (x)	0.85 (x)
4gpo	A + B	833.5	0 + 0		71	1.74 (x)	2.79 (x)
4jkv	A + B	1237.7	6 + 6		16	0.43 (b)	-1.90 (b)
3sn6	A + R	1263.1	2 + 6		103,136	0.34 (b)	-2.41 (b)

Analysis of a set of class A GPCR dimer interfaces proposed in the literature plus the proposed dimer interface for the human Smoothened receptor [PDB: 4jkv] and the β2 adrenergic receptor to G-protein interface [PDB: 3sn6]. In cases where the T4L fusion protein contributes to the interface the areas with and without (in brackets) the fusion proteins are shown. The evolutionary scores of interfaces where not enough core residues at 70% burial were present are marked with a star (*), since in such cases the evolutionary prediction can be unreliable. In all other cases next to the score we write the EPPIC classification (b for biological interface, x for crystal interface) for the particular interface, based on predefined cut-offs (0.75 for core-rim score, -1.00 for core-surface score). The EPPIC evolutionary analysis is based on UniProt version 2013_08.

In summary none of the proposed class A GPCR dimerization interfaces follow the patterns expected for high affinity biological TM interfaces in terms of geometrical packing and evolution. From this we can only conclude that if the above mentioned GPCRs do associate in oligomers, their association is likely to be weak.

Recently a structure of a class F GPCR, human Smoothened receptor [PDB: 4jkv], was solved [55] showing yet again the very well conserved 7-TM bundle. A possible dimer interface is also observed in the asymmetric unit involving helices IV and V. The structure was engineered fusing a BRIL protein N-terminally to the receptor, but BRIL does not participate in the interface. We analyzed the interface as before with the EPPIC software and find this time a very different picture than for any of the class A receptors above. In this instance the area buried in the interface is fairly large (1200 Å^2^) and more importantly each side of the interface buries 4 residues thus counting a total of 8 core residues, a good indication of a biological interface. Moreover the evolutionary indicators both agree on assigning a biological character to the interface (see Table 1). Thus in contrast to those above, we would propose a valid dimerization interface for the human Smoothened receptor. In this case, supporting evidence from FRET experiments shows that the *Drosophila melanogaster* Smoothened receptor dimerizes [56]*in vivo*. The human and fly receptors share 43% sequence identity.

As an additional control for the class A GPCR analysis we analyzed the structure of the β2 adrenergic receptor complexed with G-protein [57], where a *bona fide* biological interface exists between the receptor and the G-protein. The interface has a larger area than most of those above (1200 Å^2^) and more importantly buries 8 residues in total, typical of biological interfaces [6]. The evolutionary analysis by EPPIC shows also a very strong signal in both the core-rim and the core-surface indicators (see last entry of Table 1). It must be noted, however, that this interface, albeit a validated GPCR-partner protein interface, is not TM-spanning, which limits its value as a positive control.

## Conclusions

We have carried out a comprehensive study of all known validated TM protein-protein interfaces with high resolution and good crystallographic quality. A dataset of biological protein-protein interfaces should serve the community by facilitating further studies on membrane protein oligomerization. While we are aware that the dataset represents a small sample of the membrane protein structure space and is not bias-free, we are convinced that it contains enough data to enable useful findings.

The TM protein interfaces we studied are in broad terms not very different from those of soluble proteins: intimate packing with buried residues is needed for stable TM interfaces to form. Furthermore the residues involved in the core of the oligomerization surfaces are mostly similar in character to those in soluble proteins interfaces with a clear preference for hydrophobic ones, though alanine and glycine are to some extent overrepresented in the TM interfaces.

Importantly we conclude from our evolutionary analysis that the fingerprint of evolution can be detected in TM interfaces almost as well as in their soluble counterparts. TM interfaces possess a core of well-conserved residues that can serve to identify them when comparing against the average selection pressure of the rim of the interfaces or of the rest of the protein surface.

Additionally, we could not find significant crystallographic evidence for lipids mediating protein-protein interfaces in the transmembrane region. It must also be noted that crystallography does not seem to be ideally suited for studying membrane lipids, as their electron density almost invariably appears incomplete due to high mobility and conformational flexibility.

We also studied the proposed class A GPCR dimerization interfaces in the literature through our EPPIC method, finding that none of them seems to be a stable biological interface in light of the geometrical and evolutionary analysis. We cannot however rule out that one or more of the analyzed interfaces is a weak/transient biological interface. The recent class F GPCR structure of the human Smoothened receptor does in contrast show a clear signature of a biological interface.

## Methods

### Compilation and annotation of new reference dataset

The MPSTRUC database from Stephen White’s lab was downloaded in XML format on the 5th of October 2012. From the entries we kept those that were solved by X-ray crystallography of 3-dimensional crystals, resolution was better than 2.8 Å and Rfree below 30%. Within those constraints, we selected for further screening the best resolution representative of each cluster of identical proteins. That resulted in 69 structures from the beta class and 105 from the alpha class. We then did manual curation of each of the entries by checking the relevant literature, in order to find out whether their oligomerization state was well established and backed up by experimental data independent from crystallography. From those we could validate 3 beta monomers, 16 alpha monomers, 16 beta oligomers and 46 alpha oligomers. The 62 oligomers were then manually inspected in order to find out which of the interfaces were spanning the TM region. We checked the membrane location with the help of the OPM [58] and PDBTM [59] databases. Some of the interfaces spanned both the TM as well as the soluble regions. In those cases, interfaces that were mostly in the soluble regions were discarded.

Additional file 1 contains the full list of interfaces together with their buried areas and the EPPIC results for each of them. Additional file 2 contains the annotations and literature references with evidence of their oligomerization states.

### Interface geometry and EPPIC analysis

Interfaces were calculated with version 2.0.2 of the EPPIC package [6], using the default parameters: cofactors were considered as part of the protein surfaces for the ASA calculations whenever they were larger than 40 non-Hydrogen atoms. Interface core residues are considered those that bury more than 95% of their ASAs upon interface formation [32]. For the evolutionary predictions the version 2013_08 of the UniProt database was used. An evolutionary call could be given if at least 10 sequence homologs could be found within 60% identity of the query, or if not enough the identity cut-off was relaxed to 50%. In the evolutionary scores (core-rim and core-surface), the core residues are defined as those burying more than 70% of their ASAs upon interface formation as per EPPIC defaults.

### Statistical analysis of interface residue composition

Statistics were gathered for both our newly compiled biological TM interfaces dataset and a soluble interface dataset composed of several published datasets: DCbio [6], PLP [32], Ponstingl dimers [33] and Bahadur dimers [34]. The enrichments are defined as the log-odds ratios of frequencies in interface core residues (at 95% burial cut-off) with respect to the frequencies of all residues in the full proteins. To estimate the 95% confidence intervals in Figures 2 and 4 we used Efron’s nonparametric bootstrap [60]. A total of 5000 bootstrap samples were generated with replacement. In Figure 2 the confidence intervals were calculated from the distribution of distances to the diagonal.

The size of the dots in Figure 3 corresponds to the averaged frequency of each of the amino acids in both soluble protein set and membrane protein set. All plots were done with the open-source R statistical package [61].

The amino acids were grouped as follows:

•Hydrophobic: Ile, Leu, Met, Phe, Trp, Tyr, Val

•Polar: Asn, Gln, Ser, Thr

•Charged: Arg, Asp, Glu, Lys

•Aliphatic: Ile, Leu, Val

•Aromatic: His, Phe, Trp, Tyr

•Small: Ala, Asn, Asp, Cys, Gly, Pro, Ser, Thr, Val

•Tiny: Ala, Gly, Ser

### Lipid analysis

In order to find out lipids at interfaces the command line version of EPPIC was used and run with two different settings: 1) calculating BSAs ignoring all small molecules, 2) calculating BSAs taking molecules of more than 20 non-Hydrogen atoms as attached to their corresponding chains. Any change of interface area or interface core residues between the two runs was then inspected manually for possible lipid interactions at the interfaces.

For the Twilight analysis the version 2013-01-16 of the Twilight annotations was downloaded from the program server [44]. 11 representative PDB membrane protein structures were selected from the alpha subclass covering some of the most important groups of membrane proteins. Only those that contained some lipids and that were present in Twilight, which depends on the PDB entries being present in the EDS server [62], could be taken.

## Abbreviations

EPPIC: Evolutionary protein protein interface classifier; PDB: Protein data bank; GPCR: G-protein coupled receptor; ASA: Accessible surface area; BSA: Buried surface area; TMP: Trans membrane protein; TM: Trans membrane; FRET: Förster resonance energy transfer; LCP: Lipidic cubic phase; Sc: Shape complementarity.

## Competing interests

The authors declare that they have no competing interests.

## Authors’ contributions

JMD and GC conceived and designed the study. JMD, NB, KB and GC performed data analysis, JMD and GC wrote the manuscript. All authors read and approved the final manuscript.

## Supplementary Material

Additional file 1 **The full list of TMPbio interfaces and the EPPIC values calculated for them.** Id is the interface identifier, starting from 1 for the largest interface in crystal and higher ids for increasingly smaller interfaces. n1 and n2 are the number of homologs used to calculate evolutionary scores for each interface partner. If both are below 10, no evolutionary prediction can be made and thus a “nopred” appears for the evolutionary calls. In the evolutionary score fields (core-rim and core-surface) a few different issues (not shown) can lead to “nopred” calls, e.g. not enough core residues, too many mutations in core or rim with respect to wild type etc. Also NaNs will be present when no score can be calculated for a number of reasons. The final field contains the number of votes (each of the 3 indicators casts 1 vote) that lead to the final call. The value 0 votes means that the final call was based on applying a hard-area cut-off. All results can be visualized directly in the EPPIC web server by clicking on the provided links in the PDB code column. Click here for file

Additional file 2 **Manually curated TMPBio dataset, with experimental evidence from the literature.** Columns are: PDB code, protein name, size of validated assembly (number of subunits), the list of all biological interfaces in the protein, the list of all TM biological interfaces in the protein (with a “*” if the interface spans both transmembrane and soluble regions), the Point Group symmetry, experimental technique used to verify the oligomeric state, reference where the evidence was found (given mostly as Pubmed links) and comment containing our annotation. The table is additionally divided into subsections (with titles in bold in the name column) corresponding to the subdivisions present in Stephen White’s MPSTRUC database. All the subsection titles have been kept even when no representative PDB structure for the section was found, either because of resolution criterium or because no structure could be validated as oligomer. Experimental evidence abbreviations used: SEC size exclusion chromatography; AUC analytical gel filtration; AUC (SV) analytical ultracentrifugation sedimentation velocity; SLS, DLS, LS (static/dynamic) light scattering; MALS multi-angle light scattering; MALLS multi-angle laser light scattering; CCL chemical cross-linking; FRET fluorescence resonance energy transfer; NMR nuclear magnetic resonance; SAXS small angle X-ray scattering; MS mass spectrometry; native-PAGE native polyacrylamide gel electrophoresis; AFM atomic force microscopy. Click here for file

Additional file 3 **Twilight values for all lipids of 11 representative TM proteins.** The Real Space Correlation Coefficient is given (RSCC) and also the final Twilight assessment: Y if the molecule is below the RSCC = 0.6 threshold and thus was a Twilight positive, i.e. very likely to be wrongly modelled; N if its RSCC is above the 0.6 threshold; G if the RSCC of the molecule is above the 0.95 threshold, indicating highly confident modelling. Click here for file
