# Supplementary material for: An analysis of oligomerization interfaces in transmembrane proteins
Source: BMC Struct Biol. 2013 Oct 17;13:21. doi: 10.1186/1472-6807-13-21 (PMC4015793; doi:10.1186/1472-6807-13-21)
Supplement: Additional file 1 — The full list of TMPbio interfaces and the EPPIC values calculated for them. Id is the interface identifier, starting from 1 for the largest interface in crystal and higher ids for increasingly smaller interfaces. n1 and n2 are the number of homologs used to calculate evolutionary scores for each interface partner. If both are below 10, no evolutionary prediction can be made and thus a “nopred” appears for the evolutionary calls. In the evolutionary score fields (core-rim and core-surface) a few different issues (not shown) can lead to “nopred” calls, e.g. not enough core residues, too many mutations in core or rim with respect to wild type etc. Also NaNs will be present when no score can be calculated for a number of reasons. The final field contains the number of votes (each of the 3 indicators casts 1 vote) that lead to the final call. The value 0 votes means that the final call was based on applying a hard-area cut-off. All results can be visualized directly in the EPPIC web server by clicking on the provided links in the PDB code column. [file 1472-6807-13-21-S1.pdf]

Additional file 1

PDB	Id	BSA	n1	n2	geometry		core-rim		core-surface		final	
1a0t	1	1,967.37	55	55	23	bio	0.60	bio	-3.11	bio	3	bio
1a0t	2	1,965.86	55	55	23	bio	0.62	bio	-3.10	bio	3	bio
1a0t	3	1,964.43	55	55	23	bio	0.61	bio	-3.02	bio	3	bio
1af6	1	1,844.06	77	77	23	bio	0.60	bio	-2.76	bio	3	bio
1af6	2	1,842.20	77	77	22	bio	0.60	bio	-2.76	bio	3	bio
1af6	3	1,840.30	77	77	23	bio	0.60	bio	-2.75	bio	3	bio
1ek9	1	2,441.63	96	96	4	xtal	0.74	bio	-1.35	bio	0	bio
1ek9	2	2,438.95	96	96	4	xtal	0.69	bio	-1.44	bio	0	bio
1ek9	3	2,430.41	96	96	5	xtal	0.70	bio	-1.47	bio	0	bio
1eys	1	4,828.53	83	102	57	bio	0.68	bio	-7.74	bio	0	bio
1jb0	1	3,967.59	105	107	78	bio	0.38	bio	-5.86	bio	0	bio
1jb0	5	1,105.55	49	105	8	bio	0.66	bio	NaN	bio	3	bio
1jb0	7	915.10	22	22	4	xtal	0.75	bio	-1.51	bio	2	bio
1k4c	2	1,081.94	9	9	10	bio	0.73	nopred	-2.59	nopred	1	bio
1ldf	1	1,581.38	102	102	23	bio	0.46	bio	-2.19	bio	3	bio
1lgh	1	940.40	12	8	3	xtal	0.27	bio	-3.85	bio	2	bio
1lgh	3	939.70	12	8	3	xtal	0.27	bio	-3.91	bio	2	bio
1lgh	5	600.77	8	8	2	xtal	1.09	nopred	-0.60	nopred	1	xtal
1lgh	8	581.01	8	8	1	xtal	1.50	nopred	-0.07	nopred	1	xtal
1ppj	14	1,115.12	67	79	3	xtal	0.73	bio	-0.39	xtal	2	xtal
1q16	7	824.59	74	74	0	xtal	1.24	xtal	-0.41	xtal	3	xtal
1qd6	1	1,441.46	92	92	3	xtal	0.39	bio	-2.18	bio	2	bio
1rwt	2	995.20	119	119	10	bio	0.63	bio	-1.90	bio	3	bio
1rwt	3	979.02	119	119	9	bio	0.57	bio	-1.68	bio	3	bio
1rwt	5	962.82	119	119	9	bio	0.64	bio	-1.82	bio	3	bio
1u7g	1	1,707.86	90	90	22	bio	0.94	xtal	-1.04	bio	2	bio
1uun	1	1,892.53	29	29	9	bio	0.62	bio	-1.51	bio	3	bio
1uun	2	1,891.08	29	29	10	bio	0.56	bio	-1.60	bio	3	bio
1v54	4	2,716.39	124	46	42	bio	0.35	bio	-4.46	bio	0	bio
1v54	6	1,904.80	82	124	17	bio	0.59	bio	NaN	xtal	2	bio
1v54	8	1,810.38	102	46	21	bio	0.70	bio	NaN	xtal	2	bio
1v54	15	1,372.85	118	124	13	bio	0.73	bio	-1.38	bio	3	bio
1v54	30	910.99	46	82	6	bio	1.31	xtal	1.46	xtal	2	xtal
1v54	31	718.29	33	102	1	xtal	0.90	nopred	-1.51	nopred	1	xtal
1yc9	1	2,581.47	11	11	11	bio	0.47	bio	-1.70	bio	0	bio
1z98	1	1,749.51	118	118	26	bio	0.37	bio	-3.32	bio	3	bio
1z98	2	1,744.76	118	118	23	bio	0.40	bio	-3.52	bio	3	bio
2b2f	1	1,905.34	31	31	35	bio	0.84	xtal	-1.35	bio	2	bio
2bhw	1	915.74	119	119	7	bio	0.53	bio	-1.73	bio	3	bio
2bhw	2	915.19	119	119	8	bio	0.53	bio	-1.71	bio	3	bio
2bhw	3	910.88	119	119	7	bio	0.53	bio	-1.72	bio	3	bio
2bs2	3	2,050.12	16	16	8	bio	0.81	xtal	-1.08	bio	2	bio
2f2b	1	1,764.89	24	24	26	bio	0.63	bio	-1.13	bio	3	bio
2fgr	1	1,232.88	10	10	15	bio	0.35	bio	-3.24	bio	3	bio
2gr8	2	1,089.95	8	8	11	bio	1.04	nopred	1.66	nopred	1	bio
2gr8	3	1,087.53	8	8	9	bio	0.86	nopred	1.78	nopred	1	bio
2gr8	4	1,082.06	8	8	10	bio	0.93	nopred	2.05	nopred	1	bio
2j1n	1	1,570.40	110	110	18	bio	0.72	bio	-0.69	xtal	2	bio
2j1n	2	1,566.71	110	110	18	bio	0.78	xtal	-0.66	xtal	2	xtal
2j1n	3	1,564.96	110	110	18	bio	0.69	bio	-0.89	xtal	2	bio
2j58	1	4,294.95	107	107	15	bio	0.56	bio	-5.60	bio	0	bio
2j58	2	4,285.26	107	107	20	bio	0.51	bio	-5.82	bio	0	bio
2j58	3	4,257.43	107	107	20	bio	0.53	bio	-6.00	bio	0	bio
2j58	4	4,256.53	107	107	17	bio	0.52	bio	-5.69	bio	0	bio
2j58	5	4,255.17	107	107	20	bio	0.50	bio	-5.96	bio	0	bio
2j58	6	4,248.70	107	107	19	bio	0.55	bio	-6.04	bio	0	bio
2j58	7	4,242.90	107	107	19	bio	0.51	bio	-5.94	bio	0	bio
2j58	8	4,200.38	107	107	20	bio	0.51	bio	-5.82	bio	0	bio
2j7a	23	671.21	9	9	4	xtal	0.69	nopred	-0.14	nopred	1	xtal
2j8c	1	4,808.45	75	106	79	bio	0.52	bio	-7.99	bio	0	bio
2j8s	1	3,320.38	106	106	14	bio	0.35	bio	-2.85	bio	0	bio
2j8s	2	3,145.04	106	106	10	bio	0.32	bio	-2.76	bio	0	bio
2j8s	3	2,818.52	106	106	10	bio	0.56	bio	-2.20	bio	0	bio
2mpr	1	1,877.41	76	76	23	bio	0.57	bio	-2.74	bio	3	bio
2mpr	2	1,873.89	76	76	23	bio	0.54	bio	-2.84	bio	3	bio
2mpr	3	1,868.01	76	76	22	bio	0.54	bio	-2.82	bio	3	bio
2o4v	1	2,018.80	27	27	17	bio	0.76	xtal	-2.78	bio	2	bio
2o4v	2	2,015.21	27	27	17	bio	0.77	xtal	-2.75	bio	2	bio

continued on next page

PDB	Id	BSA	n1	n2	geometry		core-rim		core-surface		final	
2o4v	3	1,996.42	27	27	16	bio	0.75	xtal	-2.78	bio	2	bio
2o9d	1	1,829.57	101	101	30	bio	0.46	bio	-2.52	bio	3	bio
2o9d	2	1,717.59	101	101	29	bio	0.45	bio	-2.50	bio	3	bio
2qi9	1	1,882.77	94	94	6	bio	0.60	bio	-3.11	bio	3	bio
2w2e	1	2,553.53	14	14	28	bio	0.46	bio	-0.94	xtal	0	bio
2wgm	5	1,924.29	77	77	31	bio	0.81	xtal	NaN	nopred	1	xtal
2wgm	8	1,922.22	77	77	31	bio	0.80	xtal	NaN	nopred	1	xtal
2wgm	11	1,919.28	77	77	30	bio	0.86	xtal	NaN	nopred	1	xtal
2wgm	19	1,915.75	77	77	31	bio	0.84	xtal	NaN	nopred	1	xtal
2wgm	20	1,915.47	77	77	32	bio	0.79	xtal	NaN	nopred	1	xtal
2wgm	21	1,914.82	77	77	31	bio	0.83	xtal	NaN	nopred	1	xtal
2wgm	22	1,914.72	77	77	32	bio	0.85	xtal	NaN	nopred	1	xtal
2wgm	33	1,900.78	77	77	30	bio	0.80	xtal	NaN	nopred	1	xtal
2wgm	35	1,897.38	77	77	31	bio	0.80	xtal	NaN	nopred	1	xtal
2wgm	38	1,895.48	77	77	31	bio	0.80	xtal	NaN	nopred	1	xtal
2wgm	44	1,887.79	77	77	30	bio	0.79	xtal	NaN	nopred	1	xtal
2wie	1	1,909.58	103	103	35	bio	1.19	xtal	NaN	nopred	1	xtal
2wie	2	1,889.23	103	103	33	bio	1.20	xtal	NaN	nopred	1	xtal
2wie	3	1,877.42	103	103	35	bio	1.22	xtal	NaN	nopred	1	xtal
2wie	4	1,867.64	103	103	34	bio	1.21	xtal	NaN	nopred	1	xtal
2wie	5	1,856.78	103	103	33	bio	1.36	xtal	NaN	nopred	1	xtal
2wjn	1	4,693.27	18	102	57	bio	0.79	xtal	-4.95	bio	0	bio
2wlj	1	2,247.77	9	9	13	bio	0.86	nopred	-1.22	nopred	0	bio
2wlj	2	2,183.44	9	9	10	bio	0.61	nopred	-1.90	nopred	1	bio
2wsw	2	715.72	23	23	3	xtal	0.45	bio	-0.33	xtal	2	xtal
2zfg	1	1,440.50	104	104	19	bio	0.78	xtal	-1.74	bio	2	bio
3arc	1	6,054.21	101	82	111	bio	0.80	xtal	NaN	nopred	0	bio
3arc	4	3,644.40	113	82	41	bio	2.05	xtal	NaN	xtal	0	bio
3arc	5	3,250.90	101	102	29	bio	1.15	xtal	NaN	bio	0	bio
3b9w	1	1,983.85	15	15	28	bio	0.49	bio	-4.20	bio	3	bio
3c02	1	1,591.30	6	6	22	bio	0.33	nopred	-1.82	nopred	1	bio
3cx5	6	1,491.21	28	104	5	xtal	1.03	xtal	-0.49	xtal	3	xtal
3d5k	1	2,739.74	102	102	7	bio	0.59	bio	-2.27	bio	0	bio
3d5k	2	2,738.93	102	102	10	bio	0.58	bio	-2.46	bio	0	bio
3d5k	3	2,726.55	102	102	8	bio	0.65	bio	-2.37	bio	0	bio
3d9s	1	1,558.88	87	87	22	bio	0.55	bio	-1.84	bio	3	bio
3d9s	2	1,547.05	87	87	21	bio	0.62	bio	-1.68	bio	3	bio
3d9s	3	1,542.02	87	87	22	bio	0.58	bio	-1.54	bio	3	bio
3d9s	4	1,527.44	87	87	24	bio	0.53	bio	-1.75	bio	3	bio
3gd8	1	1,515.83	35	35	16	bio	0.35	bio	-2.46	bio	3	bio
3hb3	1	3,887.81	30	106	26	bio	0.53	bio	-4.90	bio	0	bio
3jqo	1	2,342.02	10	10	4	xtal	0.83	xtal	1.78	xtal	0	bio
3jqo	2	2,336.49	10	10	5	xtal	0.85	xtal	NaN	xtal	0	bio
3jqo	3	2,327.38	10	10	5	xtal	0.84	xtal	NaN	xtal	0	bio
3jqo	4	2,323.32	10	10	6	bio	0.77	xtal	NaN	xtal	0	bio
3jqo	5	2,306.63	10	10	5	xtal	0.78	xtal	NaN	xtal	0	bio
3jqo	6	2,306.03	10	10	5	xtal	0.87	xtal	NaN	xtal	0	bio
3jqo	7	2,299.34	10	10	5	xtal	0.88	xtal	NaN	xtal	0	bio
3jqo	8	2,299.00	10	10	5	xtal	0.73	bio	NaN	xtal	0	bio
3jqo	9	2,291.00	10	10	5	xtal	0.77	xtal	NaN	xtal	0	bio
3jqo	10	2,288.86	10	10	5	xtal	0.81	xtal	2.37	xtal	0	bio
3jqo	11	2,280.57	10	10	5	xtal	0.79	xtal	NaN	xtal	0	bio
3jqo	12	2,276.64	10	10	5	xtal	0.82	xtal	1.90	xtal	0	bio
3jqo	13	2,273.28	10	10	5	xtal	0.91	xtal	NaN	xtal	0	bio
3jqo	14	2,227.60	10	10	5	xtal	0.79	xtal	1.02	xtal	0	bio
3k3f	1	1,689.32	0	0	12	bio	1,000.00	nopred	NaN	nopred	1	bio
3kcu	1	1,615.51	111	111	16	bio	0.69	bio	-1.87	bio	3	bio
3kcu	2	1,581.06	111	111	16	bio	0.56	bio	-2.03	bio	3	bio
3kcu	3	1,491.53	111	111	17	bio	0.57	bio	-2.10	bio	3	bio
3kcu	4	1,487.20	111	111	17	bio	0.57	bio	-2.10	bio	3	bio
3kcu	5	1,426.85	111	111	16	bio	0.54	bio	-2.17	bio	3	bio
3kly	1	1,689.28	34	34	16	bio	0.64	bio	-1.27	bio	3	bio
3kly	2	1,646.64	34	34	17	bio	0.58	bio	-1.41	bio	3	bio
3kly	3	1,637.08	34	34	16	bio	0.57	bio	-1.45	bio	3	bio
3kly	4	1,631.44	34	34	16	bio	0.58	bio	-1.35	bio	3	bio
3kly	5	1,616.54	34	34	17	bio	0.56	bio	-1.51	bio	3	bio
3ldc	1	821.44	3	3	5	xtal	0.30	nopred	-1.62	nopred	1	xtal
3m71	1	1,490.55	11	11	10	bio	0.64	bio	-1.62	bio	3	bio
3pik	1	2,662.31	33	33	4	xtal	0.82	xtal	-0.82	xtal	0	bio
3rlf	1	3,993.60	103	89	30	bio	0.31	bio	-5.97	bio	0	bio

continued on next page

Additional file 1 - continued from previous page

PDB	Id	BSA	n1	n2	geometry		core-rim		core-surface		final	
3tdo	1	1,711.47	15	15	23	bio	0.72	bio	-2.71	bio	3	bio
3tdo	2	1,610.37	15	15	24	bio	0.63	bio	-2.94	bio	3	bio
3tdo	3	1,603.49	15	15	24	bio	0.57	bio	-3.06	bio	3	bio
3tdo	4	1,595.40	15	15	22	bio	0.62	bio	-2.92	bio	3	bio
3tdo	5	1,581.25	15	15	22	bio	0.62	bio	-2.89	bio	3	bio
3tij	1	1,259.18	105	105	10	bio	0.51	bio	-2.27	bio	3	bio
3vzt	1	1,452.94	78	78	14	bio	0.43	bio	-2.51	bio	3	bio
4a01	1	3,265.69	111	111	61	bio	0.32	bio	-5.68	bio	0	bio
4av3	1	2,838.90	10	10	47	bio	0.56	bio	-2.83	bio	0	bio
4f4s	1	1,633.16	107	107	28	bio	0.98	xtal	NaN	nopred	1	xtal
4f4s	5	1,577.73	107	107	28	bio	0.87	xtal	NaN	nopred	1	xtal
4f4s	6	1,567.80	107	107	29	bio	0.84	xtal	NaN	nopred	1	xtal
4f4s	7	1,553.88	107	107	26	bio	1.00	xtal	NaN	nopred	1	xtal
4f4s	9	1,545.24	107	107	28	bio	0.82	xtal	NaN	nopred	1	xtal
7ahl	1	2,817.31	3	3	11	bio	0.96	nopred	0.14	nopred	0	bio
7ahl	2	2,809.31	3	3	11	bio	1.07	nopred	0.83	nopred	0	bio
7ahl	3	2,793.55	3	3	13	bio	0.89	nopred	0.14	nopred	0	bio
7ahl	4	2,789.40	3	3	12	bio	0.85	nopred	0.15	nopred	0	bio
7ahl	5	2,785.36	3	3	12	bio	0.88	nopred	0.05	nopred	0	bio
7ahl	6	2,763.94	3	3	12	bio	0.90	nopred	0.16	nopred	0	bio
7ahl	7	2,745.04	3	3	12	bio	1.04	nopred	0.50	nopred	0	bio
