# Supplementary material for: An analysis of oligomerization interfaces in transmembrane proteins
Source: BMC Struct Biol. 2013 Oct 17;13:21. doi: 10.1186/1472-6807-13-21 (PMC4015793; doi:10.1186/1472-6807-13-21)
Supplement: Additional file 2 — Manually curated TMPBio dataset, with experimental evidence from the literature. Columns are: PDB code, protein name, size of validated assembly (number of subunits), the list of all biological interfaces in the protein, the list of all TM biological interfaces in the protein (with a “*” if the interface spans both transmembrane and soluble regions), the Point Group symmetry, experimental technique used to verify the oligomeric state, reference where the evidence was found (given mostly as Pubmed links) and comment containing our annotation. The table is additionally divided into subsections (with titles in bold in the name column) corresponding to the subdivisions present in Stephen White’s MPSTRUC database. All the subsection titles have been kept even when no representative PDB structure for the section was found, either because of resolution criterium or because no structure could be validated as oligomer. Experimental evidence abbreviations used: SEC size exclusion chromatography; AUC analytical gel filtration; AUC (SV) analytical ultracentrifugation sedimentation velocity; SLS, DLS, LS (static/dynamic) light scattering; MALS multi-angle light scattering; MALLS multi-angle laser light scattering; CCL chemical cross-linking; FRET fluorescence resonance energy transfer; NMR nuclear magnetic resonance; SAXS small angle X-ray scattering; MS mass spectrometry; native-PAGE native polyacrylamide gel electrophoresis; AFM atomic force microscopy. [file 1472-6807-13-21-S2.pdf]

Additional file 2- Dataset of validated TM protein interfaces

TRANSMEMBRANE PROTEINS: ALPHA-HELICAL

pdb	name	size	bio interfaces	bio TM interfaces	PG	evidence	reference	comments
	**Adventitious Membrane Proteins: Alpha-helical Pore-forming Toxins.**							No structures from this class could be validated.
	**Outer Membrane Proteins**							
2J58	Wza translocon for capsular polysaccharides	8	1-8	1-8*	C8	SDS-PAGE	17086202	A well-known octamer, SDS-stable (mentioned in the paper without the reference, seems to be an established fact). Note that the transmembrane region is formed by 8 times the C-terminal domain. The interfaces in any case go through all 3 other domains (periplasmic)
3JQO	Type IV outer membrane secretion complex	42	1-14,15-28, 29-42, 43-70	1-14*	C14	EM	19946264	A massive structure with C14 symmetry, unlikely to happen by chance in crystal and not in physiological conditions. The 14-fold assembly fits very well to the known EM maps. There is little doubt that this is biological. Interestingly this is a heterotetradecamer: a heterotrimer (proteins TraF, TraO and TraN) is repeated 14 times in C14 symmetry to assemble the pore. 1-14 interfaces are the heterologous interface of the main protein TraF C14 assembly, 15-28 the interface in the heterotrimer between TraF and TraO, 29-42 the interface in heterotrimer between TraO and TraN. 43-70 are more interfaces in AU that most likely are induced. Beyond 70 there's even more interfaces but smaller than 400A2, so we discard (as we've done in other cases). Interfaces 1-14 contain the TM part and also a periplasmic part, the rest of interfaces are not TM
	**Bacterial and Algal Rhodopsins**							No structures from this class could be validated.
	**G Protein-Coupled Receptors (GPCRs)**							No structures from this class could be validated.
	**Autonomously Folding "Membrane Proteins" (Sec-independent)**							No structures from this class could be validated.
	**Virus Coat Proteins**							No structures from this class could be validated.
	**Glycoproteins**							No structures from this class could be validated.
	**Epidermal Growth Factor Receptors**							No structures from this class could be validated.
	**Erythropoietin-Producing Hepatocellular Receptors**							No structures from this class could be validated.
	**Integrin Adhesion Receptors**							No structures from this class could be validated.
	**Histidine Kinase Receptors**							No structures from this class could be validated.
	**Immune Receptors**							No structures from this class could be validated.
	**SNARE Protein Family**							No structures from this class could be validated.
	**Channels: Potassium and Sodium Ion-Selective**							
1K4C	KcsA Potassium channel, H+ gated	4	2	2	C4	Inhibition model, geometry	1706481, 11689936 11689935	Bacterial K+ channel (Streptomyces Lividans). It seems to be a tetramer. The 1991 paper 1706481 predicts a tetramer with a complicated experiment using a known toxin inhibitor of the channel (from Drosophila) and a mutant of the channel known not to be inhibited. By modelling the system, the stoichiometry of the channel is calculated to be 4. Much later the structure came (2001) and its C4 geometry (tight helix cone formed in a 4-fold) is unlikely to have formed by chance in crystal. This 1k4c structure is crystallised with Fab fragments to obtain high resolution, a lower resolution (2.8) one without Fab is 1jvm. Both are different crystals and still conserve perfectly the same tetrameric helix cone, providing further evidence. Prokaryot and Eukaryot K channels seem to be very closely related (see 9525854)
3LDC	MthK Potassium channel, Ca++ gated	4	1	1	C4	SDS-PAGE	12037559	Archaeal K+ channel (Methanothermobacter thermautotrophicus), only transmembrane domain at high resolution. As with all other K+ channels well known to be a tetramer. The full length is seen as tetramer in SDS-PAGE (12037559). Note that the full length structure (1lnq) is quite low resolution. Also it is not clear enough whether it can be interpreted as they do in the paper. For those 2 reasons we don't use the full length for this data set, but only the high res transmembrane domain. The cytoplasmic gating

								domain has been also solved separately (2aef).
2WLJ	KirBac3.1 Inward-Rectifier Potassium channel (semi-latched)	4	1,2	1,2	C4	AFM	17936299, 16216578	The authors in first ref did AFM studies and found KirBac3.1 to be a tetramer formed by a dimer-of-dimers. This result was also found by authors in second ref who did cryo-EM on KirBac3.1
	**Channels: Other Ion Channels**							
3M71	SLAC1 anion channel, TehA homolog (wild-type)	3	1	1	C3	SEC-MALS, CCL	20981093	Trimer by both SEC-MALS and chemical cross linking. It's a structural genomics structure: a bacterial homolog (H. infuenzae) of a plant SLAC1 anion channel.
	**Cys-Loop Receptor Family**							No structures from this class could be validated.
	**Channels: Aquaporins and Glyceroporins**						11143978 10698922	All aquaporins are structurally similar and have a tetrameric assembly. They all occur as tetramers and have a rmsd of 0.5-2.5 A.
3GD8	AQP4 aquaporin water channel	4	1	1	C4	Fluorescence experiments	20071343, 7615928	Freeze free EM studies in order to study OAP association (Orthogonal Arrays of Particles, i.e. supramolecular assemblies beyond the tetramer) were reviewed by authors in second ref. Authors in first ref made Green fluorescent protein (GFP)-labeled M1 and M23 isoforms of AQP4 to address questions about AQP4 associations and OAP dynamics than cannot be addressed by available freeze-fracture electron microscopy, biochemical (native gel electrophoresis), or biophysical (single-particle tracking) methods. They also find that the 2 isoforms (differing only in a N-terminal tail) associate in heterotetramers by doing fluorescence experiments with the GFP labelled forms. They don't provide direct evidence for the homotetramer but this evidence should be enough as they are very similar.
3D9S	AQP5 aquaporin water channel (HsAQP5)	4	1-4	1-4	C4	Homology, Geometry		40% sequence id to 3gd8 above and very close structurally, plus C4 symmetry: we can safely call this a tetramer
2F2B	AqpM aquaporin water channel	4	1	1	C4	SDS-PAGE	12519768	An archaeal aquaporin. The authors here did SDS-PAGE analysis on AqpM and found that AqpM remained functional after incubations at temperatures above 80 °C and formed SDS-stable tetramers.
2O9D	AqpZ aquaporin water channel	4	1,2	1,2	C4	EM, SDS-PAGE	10518952 10518953, 9468603	AqpZ tetramer is stable in 1% SDS and runs as tetramers. EM studies also reveal tetrameric structure of AqpZ.
1Z98	SoPIP2;1 plant aquaporin (closed conformation)	4	1,2	1,2	C4	Similarity to other AQPs, Geometry		An aquaporin from spinach. Usual C4 symmetry and typical very conserved structure of aquaporins.
1LDF	GlpF glycerol facilitator channel	4	1	1	C4	EM	11265760	Negative stain electron microscopy of solubilized GlpF protein revealed a tetrameric structure of approximately 80 A side length. Scanning transmission electron microscopy yielded a mass of 170 kDa, corresponding to the tetrameric nature of GlpF.
3C02	PfAQP aquaglyceroporin	4	1	1	C4	Homology, Geometry		Homology to the other aquaporins plus usual C4 symmetry
2W2E	Aqy1 yeast aquaporin (pH 3.5)	4	1	1	C4	Homology, Geometry		Homology to the other aquaporins plus usual C4 symmetry
	**Channels : Formate/Nitrite Transporter (FNT) Family**							
3KCU	FocA, pentameric aquaporin-like formate transporter	5	1-5	1-5	C5	SEC-MALS, EM	19940917 20010838	FocA from E coli was crystallized by the authors in first ref. They get a pentameric assembly. But they do not do nay experiments to prove the pentameric assembly. Characterization of FocA from *Vibrio cholerae* (53% seq id, pentamers superpose with rmsd below 0.6) was done by authors in second ref. They did analytical size-exclusion chromatography coupled with static light scattering and refractive index techniques to get the molecular weight which corresponds to pentamer. Also the EM images suggest a pentamer.
3KLY	FocA formate transporter without formate	5	1-5	1-5	C5	SEC-MALS, EM	20010838	Characterization of FocA from *Vibrio cholerae* was done by authors. They did analytical size-exclusion chromatography coupled with static light scattering and refractive index techniques to get the molecular weight which corresponds to pentamer. Also the EM images suggest a pentamer.
3TDO	FNT3 Hydrosulphide Channel (HSC), pH 9.0	5	1-5	1-5	C5	Homology, Geometry	22407320	The structure of this HSC is also very similar to the FocA (rmsd 1.1). HSC also has a pentameric C5 assembly.
	**Channels: Urea Transporters**							
3K3F	Urea transporter	3	1	1	C3	CCL	19865084	The trimeric state of Urea transporter from *Desulfovibrio vulgaris* (dvUT) was studied by the authors with chemical crosslinking experiments. Purified dvUT was incubated with the amine-to-amine crosslinking agent disuccinimidyl glutarate at concentrations varying from 0-10 mM and then run on an SDS-PAGE gel. The peaks corresponds to the trimer of dvUT. Also, the same homotrimer was observed in a lower resolution structure obtained from the native protein, which crystallizes in a lower symmetry space group with different packing.
	**Channels: Amt/Rh proteins**							
1U7G	AmtB ammonia channel (mutant)	3	1	1	C3	SDS-PAGE, DLS, AUC, SEC	12023896	The authors here examined the quaternary structure of AmtB by SDS-PAGE, gel-filtration chromatography, dynamic light scattering and sedimentation ultracentrifugation: "The protein was resistant to dissociation by SDS and behaved as a stable oligomer on SDS-PAGE. By equilibrium desorption chromatography we determined the mass ratio of dodecyl b-D-maltoside to

								AmtB in the detergent-solubilized complex to be 1.03±0.03, and this allowed us to calculate, from analytical- ultracentrifugation data, that AmtB purifies as a trimer"
2B2F	Amt-1 ammonium channel	3	1	1	C3	Homology	16214888	Amt-1 from *Archaeglobus fulgidus* was crystallized by the authors. They found trimers in ASU. The structure of Amt-1 is very similar (rmsd ~1A) to that of AmtB from E coli. which is well characterized to be a trimer. So, we take Amt-1 also as a trimer by homology.
3B9W	Rh protein, possible ammonia or CO2 channel	3	1	1	C3	Homology	18032606, 16281947	The authors present a crystal structure of NeRh50 at 1.3 Å resolution from *Nitrosomonas europaea.* Homology modelling by authors in second ref shows that Rh is highly homologous to AmbT from E coli. which is well characterized to be a trimer. The structures are also similar. Thus we conclude by homology that NeRh is a trimer.
	**Channels: Gap Junctions**							No structures from this class could be validated.
	**Channels: Intercellular**							No structures from this class could be validated.
	**Sec Proteins**							No structures from this class could be validated.
	**Oligosaccharyl-transferases (OST)**							No structures from this class could be validated.
	**Intramembrane Proteases**							No structures from this class could be validated.
	**Membrane-Bound Metalloproteases**							No structures from this class could be validated.
	**H+/Cl-Exchange Transporters**							No structures from this class could be validated.
	**CorA Superfamily Ion Transporters**							No structures from this class could be validated.
	**Bacterial Mercury Detoxification Proteins**							No structures from this class could be validated.
	**Multi-Drug Efflux Transporters**							
2J8S	AcrB bacterial multi-drug efflux transporter (E. coli)	3	1,2,3	1*, 2*, 3*	C3	AUC-SV	17194213	This 2.54A resolution AcrB was not present in Blanco db. Added by us to have a representative of the AcrB cluster (which is in the db with a few other lower resolution members). It's a clear C3 trimer, they did AUC-SV. 2 darpins are attached to the trimer Interfaces 1,2,3 have a TM part and a soluble part.
	**Membrane-Associated Proteins in Eicosanoid and Glutathione Metabolism (MAPEG)**							No structures from this class could be validated.
	**Major Facilitator Superfamily (MFS) Transporters**							No structures from this class could be validated.
	**Solute Sodium Symporter (SSS) Family**							No structures from this class could be validated.
	**Concentrative Nucleoside Transporters (CNT)**							
3TIJ	Concentrative nucleoside transporter in complex with uridine	3	1	1	C3	CCL	22407322	To test whether vcCNT is trimeric, the authors performed structure-guided disulfide bridge cross-linking experiments. The cysteine mutants readily formed disulfide cross-linked trimers in both detergent micelles and cell membranes under oxidizing conditions.
	**Nucleobase- Cation-Symport-1 (NCS1) Family**							No structures from this class could be validated.
	**Nucleobase- Cation-Symport-2 (NCS2) Family**							No structures from this class could be validated.
	**Betaine/ Choline/ Carnitine Transporter (BCCT) Family**							
2WSW	CaiT carnitine transporter	3	2	2	C3	BN-PAGE, SEC, CCL, EM	20829798, 16365043	The pdb entry here corresponds to CaiT from Proteus Mirabilis and was crystallized by the authors in the first ref. CaiT from Proteus Mirabilis is very similar structurally and by sequence (~70% seq id) to CaiT from E. coli.<br>Authors in second ref studies the oligomeric state of CaiT from E coli. Blue native gel electrophoresis indicated that CaiT was a

								trimer in detergent solution. This result was further supported by gel filtration, cross-linking and EM studies.
	**Amino Acid/ Polyamine/ Organocation (APC) Superfamily**							No structures from this class could be validated.
	**Amino Acid Secondary Transporters**							No structures from this class could be validated.
	**Cation Diffusion Facilitator (CDF) Family**							
	**Antiporters**							
1OKC	Mitochondrial ADP/ATP Carrier	1	-	-		SEC, Negative dominance studies	6243949, 14603310, 14498831, 16226253, 17056710, 17566106	ADP/ATP carrier from beef heart mitochondria was characterized by authors in first ref. They found that triton-solubilized CAT-protein complex exists as a dimer composed of two peptide subunits. The authors in second ref crystallized the ADP/ATP carrier from bovine heart.Apparently the inhibitor atractyloside produces a difference in dimerization (see third ref): with the inhibitor the structure is homodimer, without it is monomer (both by SEC).<br>1okc here does have the atractyloside inhibitor, thus it should be a dimer. BUT all interfaces are either in upside-down or head to tail orientations. Thus unlikely that any of those are real.<br>2c3e is the same protein (with atractyloside inhibitor) in a different crystal form solved later. Both do have an interface in common: 3 of 1okc and 1 of 2c3e, but extremely small (~290A2) and in an upside-down orientation with respect to each other. Interface 3 of 2c3e does have a 2 fold parallel orientation, with 219A2 of area. They claim in paper (4th ref) that it could be the interface that is compatible with the known homodimerization, but they are not convinced themselves as they say more evidence is needed.<br>Later (ref 5) it was proven to be a monomer in detergent (by comprehensive SEC study and other techniques) and finally (ref 6) that it functions as a monomer in the membrane (by negative dominance studies).<br>The case is settled for monomer.
	**Apical Sodium-Dependent Bile Acid Transporters (ASBT)**							No structures from this class could be validated.
	**Energy-Coupling Factor (ECF) Transporters**							
3RLB	ThiT, S component of the Thiamin Transporter	1	-	-		SEC-MALLS	20218726, 21706007	The authors in first ref characterized ThiT using Size exclusion chromatography coupled to static light scattering, refractive index, and UV absorbance measurements (SEC-MALLS). The molecular mass of ThiT in the DM micelle determined by the light scattering analysis was 22.7 kDa. As the molecular mass of ThiT calculated from the amino acid sequence was 21.2 kDa, it was concluded that ThiT was monomeric in the DM solubilized state.
	**ATP Binding Cassette (ABC) Transporters**							
2QI9	BtuCD Vitamin B12 Transporter	5	1-6	1		EPR	17673622	The authors here solve the crystal structure of BtuCD in complex with BtuF. BtuF resides in the periplasmic region. But the BtuCD-F complex has substantial conformational changes as compared with the previously reported structures of BtuCD and BtuF.<br>By the Electron paramagnetic resonance (EPR) spectra studies done by the authors we can conclude that BtuCD-F chain contacts are biological. There are a total of five chains in the complex.<br>1 to 6 bio interfaces but only 1 is bio TM
3RLF	MalFGK2-MBP Maltose uptake transporter complex	5	1-6	1		SEC, CCL	21825153, 2026607	The authors in the first ref crystallized periplasmic MBP in complex with MalF, MalG and dimer of MalK from E coli. This complex was characterized by authors in second ref. They found that in all experiments, the MalF, MalG, and MalK proteins behaved as a multiprotein complex. They performed gel filtration experiment and Chemical cross-linking experimetns. Each complex contains two MalK, one MalF, and one MalG proteins.<br>1 to 6 bio interfaces but only 1 is bio TM
	**Methyltransferases**							No structures from this class could be validated.
	**Phosphoenolpyruvate-Dependent Phosphotransferases (PTSs)**							No structures from this class could be validated.
	**Superfamily of K+ Transporters (SKT proteins)**							No structures from this class could be validated.
	**Membrane-Integral Pyrophosphatases (M-PPases)**							
4A01	H+-translocating M-PPase	2	1	1	C2	RI, SEC, SDS-PAGE	10748246	The authors in the ref have listed all the methods and the respective references of the studies. The H$^+$- PPase has been strongly suggested to exist as a dimer, using Radiation Inactivation, gel permeation HPLC and SDS/PAGE analysis. Although it is not clear whether higer oligomerization is also possible. We'll take at least the dimer interface to be biological.

4AV3	Na+-translocating M-PPase with metal ions in active site	2	1	1	C2	SEC-MALLS	21664973	This is a bacterial (Thermotoga Maritima) homolog of plant (mung bean) M-PPase above (4a01) with 37% seq id. Structural conservation between the 2 is very good and also for the dimer interface providing first strong clue for dimer. The authors in first ref perform Size exclusion chromatography coupled with static light scattering to proof that a few M-PPase proteins from different organisms are dimeric, including one from Symbiobacterium thermophilum which is 42% seq id from this one. Seems to be safe to call it a dimer.
	**Bacterial V-type ATPase**							No structures from this class could be validated.
	**F-type ATPase**							
4F4S	ATP synthase (F1c10)	10	1,5,6,7,9	1, 5, 6, 7, 9	C10	CCL	10576729, 9792682, 9642286	This is the very well studied subunit c of TM domain FO of ATP synthase from yeast, a C10 ring is seen in the crystal structure (high symmetry unlikely to be an artifact). The E coli homolog was well studied with cross linking and found to have 9-12 subunits assembling in a ring in the membrane, see refs 2 and 3. Additionally another crystal form was solved for the yeast ring (3u2f, 3u2y, 3u32, 3ud0) containing the exact same C10 ring. Also the full complex of F1 together with this c10 of FO was solved (1qo1, ref 1) and shows the 10 stoichiometry. There seems to be enough evidence for this assembly to be biological.<br>The AU has 2 different half-barrels of the C10 assembly. Chains A-E are one half and chains K-O are the other. The C10 happens then on a 2 fold that completes the barrel by repeating A-E or K-O. We chose as bio TM interfaces those in the AU between K-O chains (interfaces 1,5,7,9) and the 2-fold interface between O and K (interface 6)
2WGM	Rotor (c11) of Na+-dependent F-ATP Synthase	11	5, 8, 11, 19, 20, 21, 22, 33, 35, 38, 44	5, 8, 11, 19, 20, 21, 22, 33, 35, 38, 44	C11	CCL, Homology, SDS-PAGE	9642181, 15860619, 19500592	The authors in first reference do a SPS-PAGE analysis on F1F0 ATPase from Ilyobacter tartaricus and finds that the c subunits forms a strong aggregate with the apparent molecular mass of 50 kDa which requires treatment with trichloroacetic acid for dissociation. That does not correspond to the 11x10KDa assembly seen in crystal but in any case is an indication that the protein oligomerizes very tightly.<br>The authors in second ref crystallize F0 subdomain of ATPase from Ilyobacter tartaricus and solve the crystal structure. There are 4x11-mers barrels in AU, with 2 groups of 2 stacked vertically (unlikely to be natural). Other organisms seem to have similar same ring arrangements with different numbers of subunits (see review 11893513). In any case it seems to be an established fact that this c subunit is a C11 barrel. See for instance the CCL evidence for the yeast ATP synthase above.
2WIE	Rotor (c15) of H+-dependent F-ATP Synthase of an alkaliphilic cyanobacterium	15	1,2,3,4,5	1, 2, 3, 4, 5	C15	SDS-PAGE, AFM	16170308	The oligomeric c ring of the F-ATP synthase from the alkaliphilic cyanobacterium *Spirulina platensis* was studied by the authors in this ref. They found by SDS-PAGE experiments that the enzyme moved slowly than c14 assembly suggesting that the ring contains more than 14 subunits. AFM pictures from the same authors shows that the ring contains 15 subunits.
	**P-type ATPase**							No structures from this class could be validated.
	**Phosphotransferases**							No structures from this class could be validated.
	**Hydrolases**							No structures from this class could be validated.
	**Oxygenases**							No structures from this class could be validated.
	**Oxidoreductases**							
1Q16	NarGHI Nitrate Reductase A	6	7	7	C2	SLS	12910261	The oligomerization of NarGHI was analyzed using static light-scattering chromatography. The NarGHI is a dimer of a heterotrimer and is a 'flower'-shaped structure. Static light-scattering analysis of the purified NarGHI in the presence of the detergent Thesit shows a NarGHI dimer of heterotrimers.<br>Bio interfaces are 1,2,3,4,5,6,7 but strictly transmembrane interface is only 7 (C+C chains on crystallographic 2-fold axis)
2J7A	NrfH Cytochrome C Quinol Dehydrogenase	6	2, 5, 8, 12, 14,17, 20, 23, 26, 30	23*	C2	SEC	17139260	This is a complex of NrfH (a TM chain) and NrfA (a soluble dimer): $NrfHA_2$. In crystal 2 NrfHA2 complexes (2 chains of NrfA dimer + 1 chain of NrfH) come together to form a C2 dimer super complex. The determined molecular mass of the whole NrfHA2 complex is 300 kDa by SEC, which corresponds to two $NrfHA_2$ units.<br>There are 3 copies of the super complex in the AU. We chose as reference the copy with chains A,B,C,D,E,F: A,B,D,E are the purely soluble chains; C,F are the partly TM ones.
	**Mo/Wbis-MGD Oxidoreductases**							No structures from this class could be validated.
	**Electron Transport Chain Complexes: Complex I**							No structures from this class could be validated.
	**Electron Transport Chain Complexes: Complex II**							
2BS2	Fumarate Reductase Complex	6	1-8	3	C2	SEC	10586875, 17024183, 11004459	Quinol:Fumarate Reductase complex from *W. succinogenes* was solved by the authors in second ref. Fumarate Reductase was first solved by authors in first ref. The authors in third ref study the dimer formation of Fumarate Reductase (3700 A^2) by analytical gel filtration technique and found that this dimer is apparently also present in the detergent-solubilised state of the enzyme, implying that it is unlikely to be an artifact of crystallisation.<br>Each monomeric unit is a hetero-trimer with three chains. The C2 dimer of trimers is in AU. Only chains C+F are in TM region.
	**Electron Transport Chain Complexes: Complex III (Cytochrome bc1)**							

1PPJ	Cytochrome bc1	10	1, 4, 6, 9, 10, 14, 16, 18, 20, 21, 24, 25, 31, 33, 36, 40	14*		SEC, EM	2982319, 6273583, 9204897, 16024040	Bovine cyt bc1. The authors in first ref did gel filtration experiments on Bovine heart cytochrome b-c1 complex. They found that the molecular conversion between the monomeric and dimeric state of the enzyme was reversible and dependent on the detergent concentration. The authors in the second ref did EM studies and found bc1 to be dimeric.<br>Authors in third ref claim that under the conditions of activity assays the complex is most likely in the dimeric state. We take only the contacts in one monomer to be biological (10 chains: A-J). The AU contains a dimer of 2x10 subunits.<br>As TM contacts we can take only one (with 2 copies in AU) between C+G (or P+T), it is mostly TM although there is quite some part from the smaller chain in the soluble region. All the other TM interfaces, those for chains D(Q) and E(R) with chain C(P), are much larger in the soluble region than in the TM region, so we don't take them.
3CX5	Cytochrome bc1	11	2, 3, 6, 7, 10, 17, 18, 21, 22, 24, 27, 28, 34, 36, 37, 39	6*		Homology	18390544	Yeast cyt bc1. The authors crystallize cyt bc1 complexed with cyt-c (known to be a transient interaction). The structure is similar to Bovine heart cyt bc1 complex (rmsd 1.8). Similar to previous entry we consider only monomeric units and take 9 subunits of the monomer as biological. We exclude the cyt c (chain W) which interacts only with one of the 2 monomers of the AU. Note that there's also antibody fragments in crystal (chains J(U) and K(V)).<br>As bio interfaces we take those between the first monomer (9 chains: A-I). Interface 40 (W+O) corresponds to the transient cyt-c to cyt-bc1 transient interaction.<br>As bio TM interfaces we take as before only the one that has interactions mostly in the TM region C+H (with another copy in AU N+S). The others have interactions mostly in soluble regions.
	**Electron Transport Chain Complexes: Cytochrome b6f of Oxygenic Photosynthesis**							No structures from this class could be validated.
	**Electron Transport Chain Complexes: Complex IV (Cytochrome C Oxidase)**							
1V54	Cytochrome C Oxidase, aa3	26	1, 4, 6, 8, 10, 14, 15, 18, 20, 21, 26, 29, 31, 33, 35, 37, 38 28, 30	4, 6, 8, 15*, 30, 31	C2	CCL, RI	8241183, Ref, 3017697	The authors in first ref did CCL experiments on Bovine Heart Mitochondrial Cytochrome c Oxidase in Triton X-100. Their results indicate that subunit I from each monomer provide one site of interaction between monomers in the dimeric form of the enzyme and that cytochrome c oxidase monomers may reassociate to form dimeric complexes in phospholipid vesicles.<br>Radiation Inactivation studies by authors in third ref also shows that C oxidase from Bovine heart is dimeric in nature.<br>The authors in second ref did the structure studies on Bovine Heart Mitochondrial Cytochrome and found 13 subunits in the monomer.<br>We take as bio all interfaces of first 13-mer monomer (A to M chains) (first line) and the inter-monomer interfaces (second line), discarding anything below 400A2<br>As bio TM only the subset that is mostly TM (some are mostly soluble)
3HB3	Cytochrome C Oxidase, aa3	2	1	1*		SEC	3017928, 19374884	Three different detergents CsE45, dodecyl maltoside, and Triton X-t00 have been used to study the aggregation state of Paracoccus cytochrome *C* oxidase. Three different sedimentation coefficients ranging from 4.1 to 12.2 were obtained, but in all cases the enzyme proved to be monomeric. It seems thus that, for this enzyme, the monomer (of 2 subunits) is the stable and most active form, while in the mammalian oxidase the dimer is considered to be most active.
	**Nitric Oxide Reductases**							No structures from this class could be validated.
	**Photosystems**							
1JB0	Photosystem I	36	1-6, 7, 8-20	1, 5, 7	C3	SEC, EM	Ref, 11418848	The trimeric nature of the protein was predicted by the authors in first ref. The trimeric nature was proved by HPLC gel filtration. The subunit composition was analyzed by SDS gel electrophoresis. Their PSI contained 11 subunits in a monomer. The structure described in the pdb here contains 12 subunits (an extra 35 residues X subunit), but the authors do not do any experiments to confirm that the 12th subunit also sticks together. They even call it "controversial" subunit.<br>As bio we take all interfaces in AU plus interface 7 which is the one that mediates the trimerization<br>As bio TM we take the ones that have a significant interaction at the TM region and are not mainly mediated by soluble parts or by chlorophyls/carotenoids (the structure is more of a protein+chlorophyl+carotenoids complex)
3ARC	Photosystem II	20	1, 4, 5, 7, 9, 12, 14, 15, 16, 19, 21, 22, 23, 25, 29, 31, 33, 35, 36, 37, 43, 44, 47, 48, 52, 56, 57, 59, 61, 62, 64, 67, 70, 73	1, 4, 5		Blue Native - PAGE	Ref, 21499260, 11217865, 12518057	PS II from *Thermosynechococcus vulcanus* is very similar to those from *Thermosynechococcus elongatus*. PS II in these organisms appears as a dimer of 20 subunits in the crystal. The authors in the third ref do SDS-PAGE analysis on PSII, and all the subunits break down in SDS. The authors in first ref did a BN-PAGE analysis of PS II. They found that PS II exists in equilibrium between dimer and monomer depending on the concentration of the detergent. But all 20 subunits moved together, which indicates that 20 subunits have bio contacts, while it is not so clear what to say about the dimer<br>As bio interfaces we take all in AU of first subunit (19 chains with capital letters)<br>As bio TM we take the largest mostly TM ones without taking any of the peripheral helices which are mostly mediated by chlorophyls/carotenoids
	**Light-Harvesting Complexes**							
1LGH	Light-Harvesting Complex	16	1, 3, 5, 8	1, 3, 5, 8	C8	AUC	Ref, 8736556	The structure of LH2 from Rhodospirillum molischianum is also similar to Rps. acidophila (see above), but is an octamer of heterodimers. Here also the alpha and beta apoproteins are present as homodimers. The oligomeric studies of the complex by

								authors in first ref. was done by sedimentation equilibrium experiments in the analytical ultracentrifuge. The relative molar mass of the protein/pigment complex was found to be 114 500 ± 7% (maximum error), corresponding to the (8.0 ± 0.6)-fold of that of the basic unit. The complex is thus the octamer of the basic unit (a heterodimer). The SDS-PAGE analysis and electron micrographs were studied by authors in second ref.<br>We choose chains A,B,D,E from AU as reference for building the 16-mer. Another 16-mer can be built from chains G,H,J,K.
1RWT	Light-Harvesting Complex LHC-II, Spinach Photosystem II	3	2, 3, 5	2, 3, 5	C3	SDS-PAGE, Homology	1885603, 15029188, 15719016	The authors in first ref characterized LHC II in barley. The protein was found to be trimeric by SDS-PAGE analysis. In higher plants the LHC II sequence is very conserved: it has around 90% sequence similarity in spinach, pea and barley.<br>LHC II in Barley is well characterized as trimer, so the proteins here which are structurally similar to barley should also be trimers.<br>In 1rwt there are 3 copies of the trimer in AU, thus interfaces 1-9 are bio, we take anyway chains B,G,F as reference and use only the trimer formed by interfaces 2,3,5 among them. In 2bhw there's only 1 copy in AU, thus bio interfaces are 1-3.
2BHW	Light-Harvesting Complex LHC-II, Pea Photosystem II	3	1, 2, 3	1, 2, 3	C3			
	**Photosynthetic Reaction Centers**							
2WJN	Photosynthetic Reaction Center	4	1, 2, 3, 4, 5	1		Homology	19743880, 2819866, Ref, 22439175, Ref, 10727607	The RC of *Blastochloris viridis* (2wjn) consists of four polypeptide subunits. The L and M subunits have five transmembrane helices each, and form a membrane-spanning heterodimer. And additional subunit (H) also participates with a single helix in the TM spanning region. The 4th subunit is C, which is out of the TM region. This protein has been studied extensively (20 structures in pdb).<br>The RC from RB. SPHAEROIDES contains three chains (L, M and H) which are structurally very similar to that of viridis, the extra C chain is missing here. Bactereochlorophyll molecules are present between the two chains in the center (L, M). It has been characterised extensively since the 60s-70s. Ref 6 (10727607) characterises it by AUC with a weight of 100KDa which corresponds to the observed heterotrimer in AU.<br>The RC from THERMOCHROMATIUM TEPIDUM is also structurally very similar to the viridis structure (almost identical). It also has four subunits.<br>Note that for the 3 cases we take as TM interface only 1 (L+M), interface 2 (H+M) does have a helix in the TM region but most of the interface happens in the soluble region.
2J8C	Photosynthetic Reaction Center	3	1, 2, 3	1		AUC		
1EYS	Photosynthetic Reaction Center	4	1, 2, 3, 4, 5	1		Homology		

TRANSMEMBRANE PROTEINS: BETA-BARREL

pdb	name	size	bio interfaces	bio TM interfaces	PG	evidence	reference	comments
	**Beta-Barrel Membrane Proteins: Porins and Relatives**							
2FGR	Omp32 anion-selective porin	3	1	1	C3	DLS	16434398, 9761864	Homotrimer by light scattering
2ZFG	OmpF Porin	3	1	1	C3	CCL	3013869, 18636093	
2J1N	OmpC Osmoporin	3	1,2,3	1,2,3	C3	SDS-PAGE, CL	16949612, 374117	Runs as trimer in SDS-PAGE, becomes monomer upon heating
2F1C	OmpG **monomeric** porin	1	-	-		SEC	16797588, 12899633	SEC and pore gating studies: opens and closes in one step
2MPR	LamB Maltoporin	3	1,2,3	1,2,3	C3	AUC	9102468, 21640073	*S. typhimurium* sequence. AUC data (not shown) carried out on E. coli sequence which is about 80% identical (see below). Well known to be a trimer, e.g. by EM studies
1AF6	LamB Maltoporin	3	1,2,3	1,2,3	C3	AUC	9299337, 21640073	*E. coli* sequence.
1A0T	ScrY sucrose-specific porin	3	1,2,3	1,2,3	C3	SDS-PAGE	9437428	Runs as oligomer (trimer) in SDS-PAGE, becomes monomer upon heating
1UUN	MspA mycobacterial porin	8	1,2	1*,2*	C8	SDS-PAGE	14976314, 12767342	Run in SDS-PAGE with an apparent MM of ~ 100 KDa (the monomer would be 20 KDa), this species is octamer as seen in X-ray structure.
2O4V	OprP phosphate-specific transporter	3	1,2,3	1,2,3	C3	SDS-PAGE	17187075, 2834340	Runs as trimer in SDS-PAGE, becomes monomer upon heating
3VZT	PorB outer membrane protein, native structure	3	1	1	C3	SEC	8616894	SEC measurement for both natively purified and refolded PorB.<br>Originally we had 3a2r, but it was obsoleted and replaced by 3vzt
	**Outer Membrane Carboxylate Channels (Occ)**						22272184	The improved crystal structures of Opd proteins were done by the authors in the paper. They renamed the proteins as Occ as they found that that OprD channels require a carboxyl group in the substrate for efficient transport.<br>They claim that "all channels crystallizes as monomers". But they do not give any experimental evidence for the same. Some study was done on the oligomeric state of these channels in their previous versions. The results below are on their previous research.
3JTY	BenF-like Porin (putative) benzoate channel	1	-	-		SEC-MALLS	20737437	Size Exclusion Chromatography with Multi Angle Laser Light Scattering (SEC-MALLS) were done by which authors found PflBenF to be a monomer associated with one LDAO micelle.
	**Beta-Barrel Membrane Proteins: Monomeric/ Dimeric**							
1EK9	TolC outer membrane protein	3	1, 2, 3	1*, 2*, 3*	C3	SEC, SDS-PAGE	10879525, 9044294	The study of the oligomeric state was done in the second ref. "Purified TolC subjected to gel filtration exhibited a mass of 160–

								180 kDa corresponding to a trimer of a 51.5 kDa protein plus a micelle of betaOG. When TolC from the gel filtration was mixed with SDS sample buffer without boiling and analysed by SDS–PAGE, it gave a band much greater than 100 kDa. This was replaced by a band corresponding to the 51.5 kDa TolC monomer when samples were analysed with 8 M urea included in the SDS sample buffer"
1YC9	VceC outer membrane protein	3	1	1*	C3	Geometry, homology	15684414	The authors mention that VceC is trimeric. They give the structural similarity with the ToIC (above) as the reason. But they do not do any experiments to confirm it.<br>But VceC shares the same overall fold as TolC and OprM, and the three structures can be superimposed with Cα rmsd below 2.0 Å, despite the very low degree of sequence identity (8.3%).
3D5K	OprM drug discharge outer membrane protein	3	1, 2, 3	1*, 2*, 3*	C3	Geometry, homology	20399187, 15507433	The crystal structure contains a C3 homo-trimer with huge interface areas, unlikely to be a crystal artifact. Structural comparison of OprM with TolC shows similar fold (Cα rmsd of 1.6 Å) in spite of a relatively low sequence identity (19%).
3PIK	CusC heavy metal discharge outer membrane protein	3	1	1*	C3	SEC	21249122	The crystal structure is similar to TolC with a C3 trimer. They perform gel filtration (no data shown) and found it to be a trimer.
2HDI	Colicin I receptor Cir in complex with Colicin Ia binding domain	1	-	-		AUC-SE	17464289	The authors used analytical ultracentrifugation to characterize wild-type Cir, colicin Ia, and complexes of the wild-type proteins, and both wild-type/mutant complexes. Cir and colicin Ia were observed to be monodisperse monomers, and they formed a 1:1 stoichiometric complex with high affinity.<br>Note that this monomeric membrane protein in this crystal is in complex with another protein (colicin). Thus the first interfaces are actually from the membrane protein (chain A) + colicin (chain B). Interface 4 is the first A+A xtal interface (with only 295A2)
1QJP	OmpA	1	-	-		SEC, DLS	1370823, 10764596	SEC was done by the authors in first paper finding a monomer and second paper confirms it also by dynamic light scattering.
1QJ8	OmpX	1	-	-		Homology	10545325, 1987115	OmpX was crystallized by the authors in first reference. The model was found to be very similar to OmpA which is well characterized to be monomer. The rmsd of these two structures is also very low (~2A).<br>The authors in the second ref characterized Ompx. They did SDS-PAGE analysis and found the molecular weight to be 18 kDa for a sequence of length 173. This might correspond to a monomer.<br>None of the crystal contacts is parallel to membrane normal. Also Ail homolog below is very conserved and seems to be monomeric too. Most likely a monomer.
3QRA	Ail adhesion protein	1	-	-		SEC, Homology	22078566	The authors determined the molecular weight of Ail by using gel filtration chromatography. The unheated Ail sample runs primarily as a 14 kDa, for a sequence of length 157. This corresponds to a monomer of Ail.<br>Also, Ail exhibits significant structural similarity to OmpX, with an rmsd of 1.7 Å. There is substantial sequence similarity between Ail and OmpX (the two proteins are 41% identical). Ompx is found as a monomer, which adds more evidence for the oligomeric state of Ail being a monomer.
1QD6	OmpLA (PldA) outer membrane phospholipase A monomer	2	1	1	C2	AUC, CCL	9013551, 11371166	OmpLA exists as monomer in its dormant state. In the presence of substrate and cofactor (Ca ions) they form a dimer. The dimer in presence of substrate and calcium ions was confirmed by the authors in first ref. They carried out analytical centrifugation and chemical cross linking experiments to confirm the dimeric state of OmpLA. In the inactive form OmpLA exits in an equilibrium between monomer and dimer.<br>The original entry in the table was with pdb entry 1QD5, which correspond to the monomer form. 1QD6 corresponds to the dimer form.
1P4T	NspA surface protein	1	-	-		AUC, CCL	12716881	The authors performed analytical ultracentrifugation experiments as well as chemical cross-linking. Both methods (data not shown) indicated that NspA is a monomer in a detergent-containing solution.
2WJR	NanC Porin, model for KdgM porin family	1	-	-		SEC, CCL	19796645, 11773048	NanC belongs to the family of small monomeric KdgM-related porins. The NanC was found to be monomeric by authors of the first ref in gel filtration experiments. The studies on KdgM proteins was done by authors in second ref. They found that in the case of KdgM, migration in SDS-PAGE was the same whether the samples were boiled or not. Furthermore, formaldehyde cross-linking did not show any multimerization of KdgM. So NanC seems to be a monomer. The same as with any of the KdgM family members (seems to be a well established fact)
3FID	LpxR lipid A deacylase	1	-	-		SEC	19174515	Size-exclusion chromatography showed that LpxR is most likely a monomer in solution, also based on a micelle mass for DDM.
2FCP	FhuA, Ferrichrome-iron receptor without ligand	1	-	-		SEC, AUC	9856937, 9865695, 8916906	In both studies they found a monomer in the crystal. But they do not do any further studies to confirm it. The authors in first reference say that "In contrast to the typical trimeric arrangement found in porins, FhuA is monomeric". In third reference they characterised it as monomer by SEC and AUC.
	**Outer Membrane Autotransporters**						15866036	There are two known subtypes of autotransporters: monomeric autotransporters (also referred to as classical/conventional autotransporters) and trimeric autotransporters.
1UYN	NalP autotransporter translocator domain	1	-	-		SEC-MALS, CCL	21306302	They did SEC-MALS: "SEC-MALS demonstrated that BrkAβ exists as a monomer under different detergent conditions, even for the detergent C_8E_4 that was used for crystallization"<br>Also the structures of NalP is similar to Hia (rmsd 2.6A using alignment and COOT) and EspP (full structure rmsd 3.3) (see below), providing further evidence
2GR8	Hia1022-1098 trimeric autotransporter	3	2, 3, 4	2, 3, 4	C3	SDS-PAGE	16688217	Hia was purified using a streptavidin-affinity column and then ion exchange chromatography, in each case yielding a single band on an SDS–PAGE gel corresponding to the molecular weight of a trimer. The protein is trimeric in solution.
3SLJ	EspP autotransporter, post-cleavage state	1	-	-		Blue native PAGE, AUC	16262782, 22094314	Second ref corresponds to the crystal structure of EspP. The authors in first ref did Blue Native polyacrylamide gel electrophoresis, analytical ultracentrifugation and other biochemical methods showing that EspP behaves as a compact monomer.
3AEH	Hbp (hemoglobin protease) self-cleaving autotransporter with truncated passenger	1	-	-		Homology	20615416, 15728184	The authors compare the structure to NalP and EspP autotransporters. It is very similar to EspP (rmsd 0.4). The authors also did SEC for purification (data not shown) but nothing is mentioned about the oligomeric state based on SEC.

3KVN	EstA Autotransporter, full length	1	-	-		Homology	20060837	The authors here give a full structure of the autotransporter, but the basic structure is similar to those of EspP and NalP that are well characterized, so EstA must also exist as monomer.
3ML3	IcsA autotransporter (autochaperone region only)	1	-	-		SEC	21335457	In the crystal lattice, IcsA-AC is arranged as a head-on-head dimer. In solution, the protein appears to be monomeric. IcsA-AC elutes in a single peak from a gel filtration column. The molecular mass calculated from the elution volume is 26 kDa, and the expected molecular mass of a monomer is 20 kDa.
	**Omp85-TpsB Outer Membrane Transporter Superfamily**							No structures from this class could be validated.
	**Beta-Barrel Membrane Proteins: Mitochondrial Outer Membrane**							No structures from this class could be validated.
	**Adventitious Membrane Proteins: Beta-sheet Pore-forming Toxins**							
7AHL	alpha-hemolysin	7	1, 2, 3, 4, 5, 6, 7	1*, 2*, 3*, 4*, 5*, 6*, 7*	C7	SDS-PAGE	8943190, 21280135, 6272304, 9512705	The structure was solved by the authors in first two ref. They get a heptamer in the crystal structure. SDS-PAGE analysis was done by the authors in third ref. They find the molecular weight to be of a hexamer, but there might be errors in the calculation which can easily lead to a heptamer. AFM studies were done by authors in last ref. AFM images clearly show a hexamer. In any case the extensive interfaces in crystal and amazing symmetry are unlikely to be artifacts.<br>Note that all interfaces have a TM part and a soluble part.
