# Supplementary material for: An analysis of oligomerization interfaces in transmembrane proteins
Source: BMC Struct Biol. 2013 Oct 17;13:21. doi: 10.1186/1472-6807-13-21 (PMC4015793; doi:10.1186/1472-6807-13-21)
Supplement: Additional file 3 — Twilight values for all lipids of 11 representative TM proteins. The Real Space Correlation Coefficient is given (RSCC) and also the final Twilight assessment: Y if the molecule is below the RSCC = 0.6 threshold and thus was a Twilight positive, i.e. very likely to be wrongly modelled; N if its RSCC is above the 0.6 threshold; G if the RSCC of the molecule is above the 0.95 threshold, indicating highly confident modelling. [file 1472-6807-13-21-S3.pdf]

Additional file 3

PDB	Lipid name	Chain & Residue	RSCC	Resolution	Valid
3a7k	L3P	A 331	0.254	2.00	Y
3a7k	L2P	A 295	0.324	2.00	Y
3a7k	L3P	A 298	0.333	2.00	Y
3a7k	L3P	A 296	0.384	2.00	Y
3a7k	L3P	B 331	0.388	2.00	Y
3a7k	L3P	A 297	0.425	2.00	Y
3a7k	L2P	D 293	0.428	2.00	Y
3a7k	L3P	D 330	0.429	2.00	Y
3a7k	L3P	A 301	0.435	2.00	Y
3a7k	L2P	D 294	0.447	2.00	Y
3a7k	22B	D 300	0.456	2.00	Y
3a7k	L1P	B 293	0.463	2.00	Y
3a7k	L3P	A 299	0.511	2.00	Y
3a7k	L2P	B 295	0.512	2.00	Y
3a7k	L1P	B 294	0.515	2.00	Y
3a7k	L1P	A 293	0.547	2.00	Y
3a7k	L3P	A 333	0.548	2.00	Y
3a7k	22B	A 300	0.595	2.00	Y
3a7k	L2P	B 296	0.644	2.00	N
3a7k	L2P	A 294	0.727	2.00	N
3a7k	22B	B 300	0.819	2.00	N
1xio	PEE	A 310	0.690	2.00	N
1xio	PEE	A 311	0.710	2.00	N
1xio	PEE	A 307	0.711	2.00	N
1xio	PEE	A 312	0.717	2.00	N
1xio	PEE	A 313	0.735	2.00	N
1xio	PEE	A 309	0.741	2.00	N
1xio	PEE	A 315	0.756	2.00	N
1xio	PEE	A 306	0.788	2.00	N
1xio	PEE	A 304	0.796	2.00	N
1xio	PEE	A 303	0.799	2.00	N
1xio	PEE	A 302	0.839	2.00	N
1xio	PEE	A 305	0.850	2.00	N
1xio	PEE	A 308	0.857	2.00	N
2ei4	L2P	A 280	0.606	2.10	N
2ei4	22B	A 270	0.618	2.10	N
3ddl	PX4	B1415	0.638	1.90	N
3ddl	PCW	B1416	0.642	1.90	N
3ddl	SXN	A1401	0.689	1.90	N
3ddl	SXN	B1401	0.792	1.90	N
2z73	PC1	B1004	0.836	2.50	N
1k4c	DGA	C1001	0.639	2.00	N
1okc	PC1	A 983	0.517	2.20	Y
1okc	CDL	A 802	0.614	2.20	N
1okc	PC1	A 980	0.792	2.20	N
1okc	PC1	A 981	0.807	2.20	N
1okc	CDL	A 801	0.843	2.20	N
1okc	PC1	A 982	0.859	2.20	N
1okc	CDL	A 800	0.874	2.20	N
2c3e	CDL	A 802	0.654	2.80	N
2c3e	CDL	A 800	0.819	2.80	N
2c3e	CDL	A 801	0.898	2.80	N
1ppj	PEE	D2006	0.676	2.10	N
1ppj	CDL	P3003	0.798	2.10	N
1ppj	CDL	D2003	0.812	2.10	N
1ppj	PEE	Q3006	0.857	2.10	N
1ppj	CDL	G2004	0.861	2.10	N
1ppj	CDL	T3004	0.889	2.10	N
1ppj	PEE	P3007	0.916	2.10	N
1ppj	PEE	C2007	0.940	2.10	N
3arc	DGD	d 755	0.360	1.90	Y
3arc	DGD	D 755	0.388	1.90	Y
3arc	LMG	Z 784	0.482	1.90	Y
3arc	LMG	z 784	0.540	1.90	Y
3arc	LMG	C 776	0.558	1.90	Y
3arc	LHG	e 772	0.610	1.90	N
3arc	SQD	B 668	0.613	1.90	N
3arc	SQD	d 768	0.623	1.90	N

continued on next page

pdb	lipid name	chain & residue	RSCC	resolution	valid
3arc	LMG	c 776	0.646	1.90	N
3arc	SQD	a 667	0.682	1.90	N
3arc	LHG	E 772	0.701	1.90	N
3arc	SQD	L 668	0.707	1.90	N
3arc	SQD	A 667	0.736	1.90	N
3arc	SQD	D 768	0.755	1.90	N
3arc	LMG	a 751	0.772	1.90	N
3arc	LMG	A 751	0.786	1.90	N
3arc	LMG	d 692	0.860	1.90	N
3arc	LMG	c 729	0.868	1.90	N
3arc	LMG	C 729	0.870	1.90	N
3arc	LMG	D 692	0.893	1.90	N
3arc	LMG	B 669	0.892	1.90	N
3arc	SQD	a 659	0.907	1.90	N
3arc	SQD	A 659	0.905	1.90	N
3arc	DGD	c 661	0.910	1.90	N
3arc	DGD	c 660	0.910	1.90	N
3arc	DGD	C 660	0.920	1.90	N
3arc	LMG	b 669	0.926	1.90	N
3arc	LHG	D 714	0.924	1.90	N
3arc	LHG	d 714	0.924	1.90	N
3arc	DGD	H 663	0.927	1.90	N
3arc	DGD	h 663	0.931	1.90	N
3arc	LHG	l 694	0.942	1.90	N
3arc	DGD	C 657	0.944	1.90	N
3arc	LHG	D 664	0.943	1.90	N
3arc	DGD	C 661	0.947	1.90	N
3arc	DGD	c 657	0.946	1.90	N
3arc	LHG	d 664	0.952	1.90	G
3arc	LHG	L 694	0.955	1.90	G
3arc	LHG	d 702	0.960	1.90	G
3arc	LHG	D 702	0.972	1.90	G
1rwt	DGD	G9632	0.883	2.72	N
1rwt	DGD	A 632	0.882	2.72	N
1rwt	DGD	B2632	0.889	2.72	N
1rwt	DGD	D5632	0.889	2.72	N
1rwt	DGD	H6632	0.890	2.72	N
1rwt	DGD	H7632	0.890	2.72	N
1rwt	DGD	E4632	0.897	2.72	N
1rwt	DGD	I8632	0.897	2.72	N
1rwt	DGD	B1632	0.902	2.72	N
1rwt	DGD	D3632	0.902	2.72	N
1rwt	LHG	A 630	0.923	2.72	N
1rwt	LHG	G6630	0.925	2.72	N
1rwt	LHG	B1630	0.928	2.72	N
1rwt	LHG	D3630	0.930	2.72	N
1rwt	LHG	E4630	0.930	2.72	N
1rwt	LHG	J9630	0.931	2.72	N
1rwt	LHG	H7630	0.935	2.72	N
1rwt	LHG	C2630	0.933	2.72	N
1rwt	LHG	F5630	0.935	2.72	N
1rwt	LHG	I8630	0.934	2.72	N
